# Advances and challenges in immunoPET methodology

**DOI:** 10.3389/fnume.2024.1360710

**Published:** 2024-02-19

**Authors:** Philipp Mohr, Joyce van Sluis, Marjolijn N. Lub-de Hooge, Adriaan A. Lammertsma, Adrienne H. Brouwers, Charalampos Tsoumpas

**Affiliations:** ^1^ Department of Nuclear Medicine and Molecular Imaging, University Medical Center Groningen, University of Groningen, Groningen, Netherlands; ^2^ Department of Clinical Pharmacy and Pharmacology, University Medical Center Groningen, University of Groningen, Groningen, Netherlands

**Keywords:** monoclonal antibody, immunoPET, quantification, kinetic modeling, zirconium, imaging, PET/CT

## Abstract

Immuno-positron emission tomography (immunoPET) enables imaging of specific targets that play a role in targeted therapy and immunotherapy, such as antigens on cell membranes, targets in the disease microenvironment, or immune cells. The most common immunoPET applications use a monoclonal antibody labeled with a relatively long-lived positron emitter such as ^89^Zr (*T*_1/2_ = 78.4 h), but smaller antibody-based constructs labeled with various other positron emitting radionuclides are also being investigated. This molecular imaging technique can thus guide the development of new drugs and may have a pivotal role in selecting patients for a particular therapy. In early phase immunoPET trials, multiple imaging time points are used to examine the time-dependent biodistribution and to determine the optimal imaging time point, which may be several days after tracer injection due to the slow kinetics of larger molecules. Once this has been established, usually only one static scan is performed and semi-quantitative values are reported. However, total PET uptake of a tracer is the sum of specific and nonspecific uptake. In addition, uptake may be affected by other factors such as perfusion, pre-/co-administration of the unlabeled molecule, and the treatment schedule. This article reviews imaging methodologies used in immunoPET studies and is divided into two parts. The first part summarizes the vast majority of clinical immunoPET studies applying semi-quantitative methodologies. The second part focuses on a handful of studies applying pharmacokinetic models and includes preclinical and simulation studies. Finally, the potential and challenges of immunoPET quantification methodologies are discussed within the context of the recent technological advancements provided by long axial field of view PET/CT scanners.

## 1 Introduction

In a broader sense, immunoPET means imaging of specific targets that play a role in targeted therapy and immunotherapy, such as antigens on cell membranes, targets in the disease microenvironment, or immune cells by using positron emission tomography (PET) (1–7). Most commonly the term “immunoPET” is used for PET imaging with intact monoclonal antibodies (mAbs) or smaller mAb-based constructs labeled with a relatively longer-lived positron-emitting radionuclide such as ^89^Zr (8, 9). There is an increase in the development of mAb drugs (10), which are widely used in oncology but also in infectious and inflammatory diseases. These drugs are very target specific and accomplish their effects by directly acting on their target or indirectly by activating the immune system (11). Similarly, immunoPET has its main applications in oncology, but other clinical applications such as infectious applications, such as for infectious and inflammatory diseases, are also under investigation (12–17).

In general, the non-invasive, whole body *in vivo* information obtained through immunoPET can provide important information for drug development and has the potential to support patient selection and treatment decisions using a personalized, theragnostic approach (18), e.g., imaging with a radiolabeled mAb followed by treating with an antibody-drug-conjugate (ADC) or using radioimmunotherapy (RIT). In this respect, immunoPET is reminiscent of the term “magic bullet”, an idea first proposed by Paul Ehrlich (19), who envisioned that a diagnostic agent selectively targeting a disease could be combined with a therapeutic agent for treatment, selectively destroying diseased cells while sparing normal, healthy tissue.

The primary goals of immunoPET are to visualize the biodistribution of the radiolabeled tracer and to characterize target tissues with respect to the presence or absence of receptors for the ligand under investigation. Beyond visualization, radiotracer uptake can be expressed quantitatively and thus immunoPET potentially provides a quantitative whole-body image of disease-associated target expression. This would be particularly useful as intra- and interlocal heterogeneity cannot be detected with locally obtained biopsies. Ideally, PET imaging would provide a metric that reflects the amount of specific binding of the molecule to its receptors. However, this is challenging due to various nonspecific factors that affect tracer uptake, high physiological variability within and between tumors (or other diseased tissues) in terms of vascularity, vascular permeability, interstitial pressure, blood flow, and other factors (20, 21). Regarding the potential of immunoPET to predict response to targeted or immunotherapy, there are equivocal results in the literature: some studies demonstrated a correlation between PET uptake and treatment response, while others failed to do so (see section 3.1.1.3).

From a PET methodology perspective, imaging and quantification protocols are an important factor in determining how well immunoPET can assess target engagement and thus how well it can guide therapy and ultimately benefit the patient. Therefore, the main objective of this narrative review is to explore the role of PET imaging and data analysis methodology in the context of immunoPET literature. The first part of this review summarizes the vast majority of studies that apply semi-quantitative methodologies, including study design aspects such as the use of cold dose, imaging time points, and correlation with biopsies and treatment response. In addition, differences in imaging methodologies between intact mAbs and smaller mAb based constructs, as well as the use of different radionuclides are highlighted. The second part focuses on the few studies that went beyond semi-quantitative methodologies and investigated pharmacokinetic modeling approaches, including preclinical and simulation studies. Finally, the review discusses the opportunities and challenges for improving immunoPET quantification methodologies, considering static vs. dynamic PET acquisitions, dual tracer approaches, and the role of the recently introduced high-sensitivity long axial field of view (LAFOV) PET systems.

To find relevant articles, the search engines PubMED, Embase and Web Of Science Core Collection were consulted until January 6, 2024. Despite being a narrative review, the search strategy and further details on all clinical studies included in this review can be found in the Supplementary Materials.

## 2 Background

### 2.1 Antibodies as PET probes

The use of mAbs in nuclear imaging started with “radioimmunodetection” (22, 23) using gamma cameras and later SPECT imaging by labeling mAbs with ^123^I, ^111^In, or ^99m^Tc (24–27). Subsequently, the interest of the nuclear medicine community shifted towards PET radionuclides like ^64^Cu, ^89^Zr, or ^124^I (28–30), as immunoPET images allow for higher spatial resolution, more accurate quantification, and often better target-to-background ratios (TBR) compared with immunoSPECT (31–33).

Beyond the conventionally used IgG molecules, other categories include multispecific mAbs that bind to more than one target and smaller mAb based constructs (8, 9). These smaller constructs have been used to accelerate the clearance of unbound radiotracer from the circulation and background tissues and can sometimes achieve higher TBR. In addition, small antibody fragments can penetrate tumors more efficiently and homogeneously (34). A second important aspect is whether the ligand binds to only one type of receptor on the tumor cell or a specific receptor in the micro-environment of tumors [e.g., vascular endothelial growth factor receptor (VEGF)] or whether it targets immune cells (e.g., T-cells) or, in the case of multispecific mAbs, both tumor and immune cells. Radiolabeling of these multispecific mAbs is particularly interesting to study their pharmacological behavior *in vivo*, although quantification of the targets is challenging as the contribution of each target to the PET signal is unknown.

From a PET methodology perspective, it is important to understand that the size of the targeting vectors is critical to their pharmacokinetics. Molecular sizes of immunoPET tracers range from 6 kDa for the smallest constructs (affibodies) up to 150 kDa for intact mAbs with several smaller mAb-based constructs in between. While smaller molecules are rapidly removed via renal filtration, the larger ones are excreted via the hepatobiliary route. In general, an increase in molecular radius leads to a decrease in plasma clearance (35). As a result, the half-life in the bloodstream for these different molecules can range from less than 1 h up to several weeks. An overview of different mAb-based constructs and their size, biological/pharmacokinetic (PK) half-lives, and elimination pathways is provided in Table 1. This table does not claim to be exhaustive, as further engineered antibody fragments are currently being developed. In addition, smaller artificially produced peptides binding to similar targets exist, which are beyond the scope of this overview.

**Table 1:** Antibodies and antibody based constructs used for immunoPET. Adapted from (36).

Name	IgG	F(ab’)_2_^a^	Minibody	F(ab)^b^	scFv^c^	sdAb^d^/Nanobody	Affibody
Size	150 kDa	110 kDa	75 kDa	≈50 kDa	≈25 kDa	15 kDa	6 kDa
PK half-life	Few days to weeks	≈24 h	Few hours	≈4 h	≈1 h	≈1 h	<1 h
Elimination	Liver	Liver	Liver	Kidney	Kidney	Kidney	Kidney

^a^ *F*(ab)_2_: fragment antigen-binding with two antigen-binding regions.

^b^ *F*(ab): smaller fragment with one antigen-binding region.

^c^ scFv: single-chain fragment variable.

^d^ sdAb: single-domain antibody.

The half-life of the radionuclide should be compatible with the kinetics of the tracer. Longer-lived radionuclides like ^89^Zr and ^124^I are primarily used for intact mAbs. ^64^Cu has an intermediate half-life and may be used for both intact mAbs and smaller constructs. The shorter lived ^18^F or ^68^Ga are more suitable for the smaller mAb-based constructs like single-domain antibodies (sdAbs) or affibodies. Using longer-lived radionuclides limits the amount of activity that can be injected due to the radiation dose, but it enables a later immunoPET imaging time-point, giving the tracer more time to accumulate in the target regions and decreasing non-specific uptake in background tissue, which might lead to better lesion detectability. On the other hand, both the reduced injected activity and delayed imaging also lead to more noisy images compared with conventional PET.

Another important feature of the positron emitter is its behavior upon internalization following binding to the target antigen. For example, after internalization of a ^124^I-labeled mAb-antigen construct, the construct will be degraded in the lysosomes leading to free ^124^I that can rapidly leave the tissue and subsequently gets excreted renally. On the other hand, when radiometal-labeled mAbs (like ^89^Zr-mAbs) are processed, the positron emitters are trapped intracellularly in the lysosomes (37). The radionuclide remains in the cell after internalization and degradation, leading to accumulation of the signal over time. One disadvantage with radiometal-labeled mAb-based constructs is high accumulation of radioactivity in the liver or kidney due to non-specific binding to Fc receptors, which can hinder the detection of metastases in these regions (38). Table 2 shows useful radionuclides for immunoPET imaging with their half-life, positron emission intensity, positron energy, main other emission type, and whether they are radiometals or halogens. The most frequently used radionuclides are ^89^Zr, ^124^I, ^64^Cu mainly for intact mAbs and ^18^F and ^68^Ga for small mAb-based constructs, whereas ^76^Br, ^86^Y and ^44^Sc are used less frequently mainly in preclinical research.

**Table 2:** Radionuclides for immunoPET imaging, their half-lives and other characteristics. Adapted from (36).

Isotope	*T*_1/2_	*β*^+^ intensity (mean energy)	Main other emission type (energy, intensity)	Chemical/residualizing
^68^Ga	67.7 min	88.9% (829.5 keV)	*γ* (1,077 keV, 3.22%)	Metallic/Yes
^18^F	109.8 min	96.7% (249.8 keV)	/	Halogen/No
^44^Sc	3.97 h	94.3% (632.0 keV)	*γ* (1,157 keV, 99.9%)	Metallic/Yes
^64^Cu	12.7 h	17.5% (278.0 keV)	*γ* (1,345.77 keV, 0.472%) *β*^−^ (190.7 keV; 38.5%)	Metallic/Yes
^86^Y	14.7 h	31.9% (660.0 keV)	*γ* (637 keV, 32.6%) *γ* (1,076 keV, 82.5%) *γ* (1,153 keV, 30.5%) *γ* (1,920 keV, 20.8%)	Metallic/Yes
^76^Br	16.2	55.0% (1,180 keV)	*γ* (559 keV, 74.0%) *γ* (657 keV, 15.9%) *γ* (1,216 keV, 8.8%) *γ* (1,854 keV, 14.7%)	Halogen/No
^89^Zr	78.4 h	22.7% (395.5 keV)	*γ* (909 keV, 99% *T*_1/2_ = 15.66 s)	Metallic/Yes
^124^I	100.2 h	22.7% (818.5 keV)	*γ* (602 keV, 62.9%) *γ* (722 keV, 10.3%) *γ* (1,509 keV, 3.3%) *γ* (1,690 keV, 11.1%)	Halogen/No

### 2.2 Uptake mechanisms of immunoPET tracers

To find an accurate imaging metric for target engagement from immunoPET, it is necessary to understand the nature of PET uptake visible in the images and to validate the image quantification methodology. Uptake mechanisms depend on the individual immunoPET tracer, are relatively complex and not fully known (11). Nevertheless, a general overview is provided here, focusing on intact mAbs.

**Figure 1:**
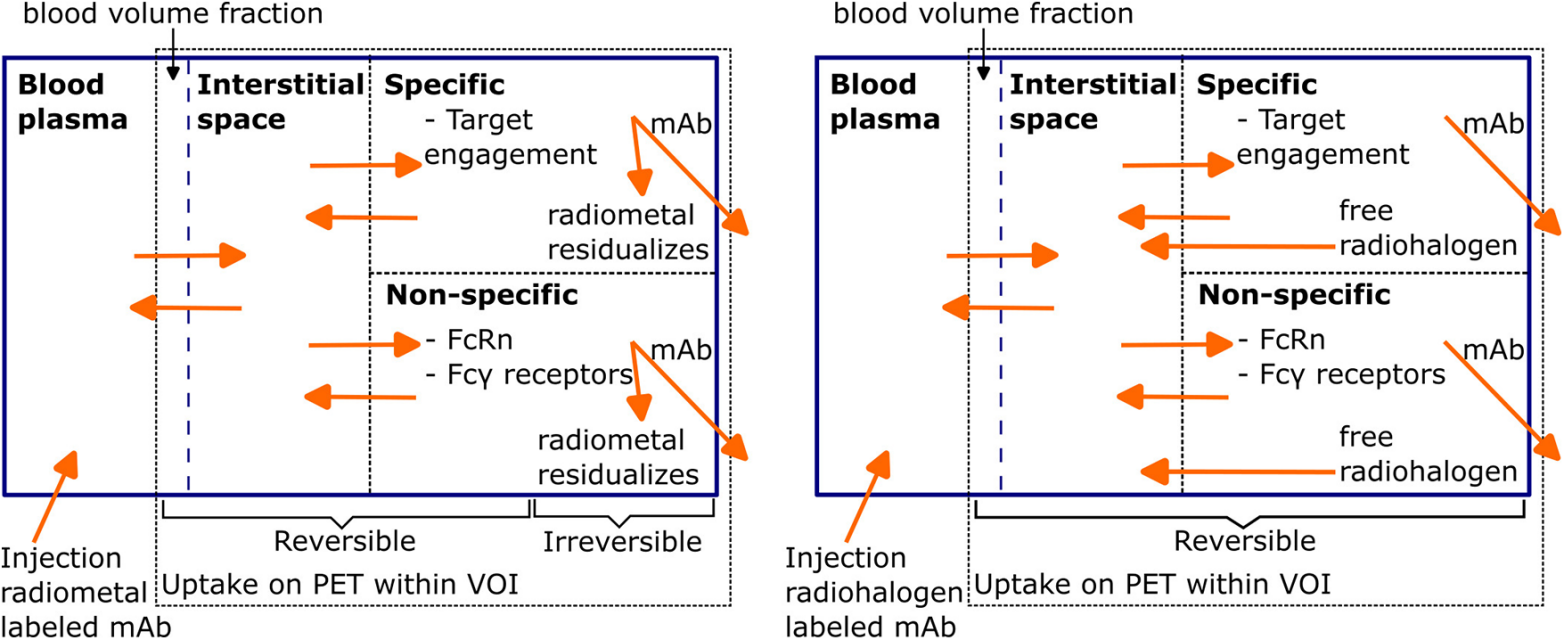
Schematic representation of PET uptake components for radiometal and radiohalogen labeled mAbs. Radiolabeled mAbs are injected into the blood and, after initial distribution, are reversibly present inside the blood volume fraction and the interstitial space of the tissue. Subsequently, specific (target engagement) and non-specific binding (Fc receptors) processes can occur, both reversibly and irreversibly. After irreversible binding of the radiolabeled mAb, the mAb-antigen construct gets internalized and degraded, after which free radiometal atoms stay inside the cell and radiohalogen atoms can leave the cell. Redrawn from Wijngarden et al. (39) (CC BY 4.0 License).

In line with the term “magic bullet”, an ideal mAb for immunoPET imaging would solely bind to the specific disease-related target, and there would be no uptake in other healthy tissues. However, most mAbs also show considerable uptake in healthy tissues due to several reversible and irreversible uptake components, which are schematically represented in Figure 1 for radiometal and radiohalogen labeled mAbs. After intravenous administration into the blood, mAbs distribute throughout the body and are mostly present in the blood volume and the interstitial space of the tissue. The so-called blood volume fraction refers to the proportion of the volume of interest (VOI) that consists of blood. Radiolabeled mAbs in the blood volume fraction and the interstitial space resemble the main part of the nonspecific uptake of mAbs in tissues, especially during the first period after injection.

Due to their size, mAbs only have a limited ability to distribute from blood to tissue by diffusion, and extravasation occurs mainly by convection or via receptor-mediated endocytosis (11, 40). The role of diffusion may be more significant in “leaky” tumor vessels (41) with increased permeability. Nonetheless, the distribution mechanisms are slow and volumes of distributions are generally low (42). mAb distribution in the tumor center is limited and overall the distribution within tumor tissues is heterogeneous (43). In contrast, smaller antibody-based constructs can cross the blood-tissue barrier more easily, which is desirable for tumor targeting (44).

mAbs in the interstitial space may enter cells via receptor-mediated endocytosis via Fc-*γ* receptors by fluid phase endocytosis or via the desired target interaction when bound to a membrane antigen (42). After internalization, the radiolabeled mAb-antigen constructs gets degraded in the lysosomes, subsequently the radionuclide either stays in the cells, which is the case for radiometals like ^89^Zr, or can leave the cell, as in the case of a radiohalogen like ^124^I as described before. The nonspecific processes of binding to Fc-*γ* receptors (45) on immunological cells or neonatal Fc-receptors (FcRn) within endothelial cells also contribute to the PET signal on top of the actual specific antigen engagement. In case of binding to FcRn, FcRn-bound mAbs are brought back into the circulation or the interstitial space. Engineered immunome tracers with a reduced or eliminated Fc region aim to minimize the non-specific interactions with FcRn and Fc-γ receptors.

For mAbs targeting antigens located on cells, the target-mediated elimination after internalization is probably the dominant elimination route. Other reported elimination routes of mAbs include nonspecific endocytosis and proteolysis by the liver and the reticuloendothelial system (11), where the phagocytic cells of the immune system such as macrophages and monocytes play a role in the elimination of mAbs.

Another concept to be introduced here is the so-called “antigen sink”. If binding sites are present in larger amounts in other tissues, such as liver or spleen, than in the actual target tissue, these organs may serve as a sink, leading to the need for a higher cold dose co-injection compared with those of target antigens that are only expressed in very specific regions.

Both linear and nonlinear eliminations have been reported for mAbs, probably caused by target-mediated disposition. Pharmacokinetics can sometimes be described by linear clearance, which may be because the concentration does not saturate the target antigen or that the target-mediated clearance has a less prominent role compared with other elimination pathways. Smaller molecules, like Fab fragments, are eliminated more rapidly than intact mAbs due to the lack of the Fc part and hence protection by FcRn. In addition, they can be excreted to some extent by the kidneys (11).

Apart from the mechanisms mentioned above, several other factors are important; they are, however, beyond the scope of this review, and the interested reader may find more information in (11, 42). It should be noted that in an immunoPET imaging study, the mechanisms described apply to both the labeled and unlabeled mAb, which often accompanies the tracer dose and affects visualization and quantification, as will be described later.

### 2.3 PET quantification in general

In most cases, PET images are analyzed semi-quantitatively providing a relative measurement in contrast to absolute quantification (46). In semi-quantitative analyses, the Standardized Uptake Value (SUV) is commonly used for measuring the uptake of a tracer (47, 48), which is the decay-corrected measured activity concentration (AC) normalized to the injected activity and the body weight or sometimes the lean body mass (LBM) of the patient. Although the use of SUV as a measure of relative uptake makes a comparison between patients easier, there is no universally accepted threshold value for characterization a region as target-positive or not, partly due to the high degree of variability and sources of error (48, 49).

Other frequently reported parameters are relative uptake ratios, where the activity concentration or SUV of the target tissue is divided by the corresponding value of another region, mostly a blood pool region on the image or a blood sample. In this way, parameters like tumor- or (more generally) target-to-blood ratios can be defined and abbreviated as TBR or, if normalized to plasma activity, TPR. Sometimes the liver or other regions are used as a background region and the same acronym TBR (tumor-to-background) may be used. If not mentioned otherwise, in the present article TBR refers to target-to-blood ratio.

The semi-quantitative metrics can be reported in terms of the maximum, peak or mean value determined in a VOI. SUV_max_ is the voxel with the maximum uptake in the VOI, and SUV_peak_ is often defined as a 1 mL spherical VOI that results in the largest mean value, although different definitions are in use (50). SUV_mean_ is the mean value of a VOI, which highly depends on the VOI definition. All PET measurements and reported parameters are subject to noise and variability due to count statistics of PET measurements. For instance, it has been shown that SUV_max_ is sensitive to noise while SUV_peak_ is less so (51, 52), which is particularly important for low count applications like ^89^Zr-immunoPET, especially at later time-points.

The definition of a VOI is performed either manually or automatically using data-driven approaches using VOIs based on PET or CT images in order to improve the reproducibility of PET quantification (53, 54). Common PET data-driven approaches include 3D isocontours at 41% or 50% of the maximum pixel value (49, 55). However, these also are sensitive to noise. Automatic VOIs are not always possible if there is high background or a high uptake area near the region of interest. Therefore, visual inspection of semiautomatic VOIs is necessary. In case of tracer uptake heterogeneity, VOIs may only delineate part of the lesion. Advanced tumor segmentation methods are beyond the scope of this paper. Finally, it should be noted that regions of size similar to the spatial resolution of the scanner suffer from partial volume effects (PVE), which leads to bias in uptake measurements (56).

Another crucial aspect is harmonization of PET measurements between different scanners and in particular in multicenter studies. This can be achieved by developing guidelines on how to acquire and how to interpret and report results of a scan. From a technical point of view, these guidelines include PET and CT acquisition and reconstruction protocols, metrics used for tumor uptake, and quality control and inter-institutional PET/CT system performance harmonization. Makris et al. (57) and Kaleep et al. (58) have investigated the feasibility of quantitative accuracy and harmonized image quality using ^89^Zr PET/CT in multicenter studies and concluded that semi-quantitative accuracy and harmonized image quality can be reached in ^89^Zr PET/CT multicenter studies provided that scanner acquisitions and reconstructions are harmonized. Similar efforts for quality control and harmonization could also be considered for immunoPET studies labeled with other radionuclides like ^124^I or ^64^Cu.

### 2.4 PET kinetic modeling

The PET signal in a VOI at a certain time post injection (p.i.) does not only reflect specific binding of the tracer but also contains several other components, as described earlier when summarizing uptake mechanisms of immunoPET tracers. Kinetic modeling approaches have the potential to distinguish between the different uptake components and thereby provide a metric that is better representative for specific target engagement than a SUV determined with a static scan.

To apply kinetic modeling, PET uptake needs to be measured at several time points, or for tracers with relatively fast kinetics, a dynamic PET scan needs to be acquired, leading to the measurement of time-activity curves (TACs). In addition, a so-called arterial input function (AIF) is needed, which represents the amount of tracer in plasma that is available for the tissue. The gold standard to obtain the AIF is arterial blood sampling. To avoid blood sampling, information about the AIF can also be derived directly from PET images [so-called image derived input function (IDIF)], by measuring the image TAC in a blood pool region (59–61). This technique needs to be validated against conventional blood sampling, as was recently presented for two ^89^Zr-mAb tracers (62).

**Figure 2:**
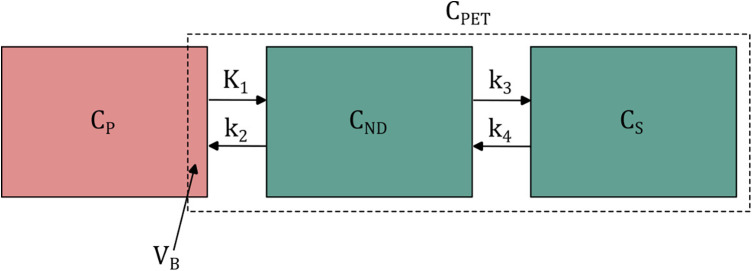
Schematic illustration of the two-tissue compartment model. $C_{P}$, $C_{\mathrm{ND}}$ and $C_{S}$ represent arterial plasma, non-displaceable tissue and specific tissue concentrations. $K_{1}$ to $k_{4}$ are rate constants for the transport between compartments. $V_{B}\;$ is the fractional blood volume within the PET region of interest. Its contribution to the total signal $C_{\mathrm{PET}}$ depends on the arterial whole blood concentration. For simplicity, however, only the arterial plasma compartment is shown.

The most commonly used tracer kinetic model is the two-tissue compartment model (2TCM) (63, 64), which is illustrated in Figure 2. Including the blood volume fraction $V_{B}$ in the VOI, the total signal $C_{\mathrm{PET}}(t)$ measured by the PET scanner is described as in Equation 1:

(1) $$C_{\mathrm{PET}}(t)=(1-V_{B})\cdot C_{T}(t)+V_{B}\cdot C_{A}(t)$$

where $C_{A}(t)$ represents the arterial whole blood concentration and $C_{T}(t)$ the tissue signal as a result of the plasma concentration, which is given by Equation 2:

(2) $$C_{T}(t)=C_{\mathrm{ND}}(t)+C_{S}(t)$$

where $C_{\mathrm{ND}}(t)$ and $C_{S}(t)$ represent concentrations in non-displaceable and specific compartments, respectively. The standard two-tissue compartment model contains four rate constants $K_{1}$ to $k_{4}$, but for tracers that are trapped in the final compartment, such as radiometal-labeled mAbs, it is reduced to an irreversible two-tissue compartment model ($k_{4}=0$). From these rate constants, the parameter $K_{i}$, the net influx rate of the tracer, can be calculated from $K_{i}=K_{1}\cdot k_{3}/(k_{2}+k_{3})$.

The rate constants are related to physiological/pharmacological parameters, in particular $K_{1}=E\cdot F$, where *E* is extraction fraction and F blood flow. *E* itself depends on *F* as described by the Renkin-Crone equation: $\;E=1-e^{-\mathrm{PS}/F}$. Since permeability surface products PS are generally low for intact mAbs, $K_{1}$ probably is dominated by PS as demonstrated in physiologically based pharmacokinetic (PBPK) models (65, 66). Effects of *F* become more relevant for smaller mAb based constructs. $k_{3}$ mainly depends on the available number of receptors, which is the number of total receptors minus the number of blocked receptors, e.g., by cold mAb dose.

In addition to conventional PET kinetic modeling, the PBPK models mentioned previously, often used in non-radiopharmaceutical drug development, can also be applied to improve understanding of biodistribution and kinetics of immunoPET tracers (67). In contrast to PET kinetic models, these models follow a more complex approach based on drug- and system-specific knowledge and are also valid for therapeutic doses. While they allow for more detailed simulation or fitting, they require more prior information and assumptions.

It can be difficult to fit 2TCM and especially PBPK models to immunoPET TACs due to the number of parameters that need to be fitted in combination with the slow kinetics requiring multiple (sparsely sampled) scan time points. On the other hand, the Patlak graphical model (68) is an alternative, simplified analysis method suitable for irreversible tracer kinetics. It is particularly interesting for immunoPET with larger molecules that involves imaging on different days because a few sampling points are enough to fit the linear model given by Equation 3:

(3) $$\frac{C_{\mathrm{PET}}(t)}{C_{P}(t)}=K_{i}\frac{\int_{0}^{t}C_{P}(\tau )d\tau }{C_{P}(t)}+V_{T}$$

where $C_{P}(t)$ is the arterial plasma input function, $C_{\mathrm{PET}}(t)$ the tissue TAC in the PET frames, $\int_{0}^{t}C_{P}(\tau )d\tau$ the integral of the input function from injection to time *t*, $K_{i}$ the slope, which is identical to the influx rate mentioned above, and $V_{T}$, sometimes called initial volume of distribution, is related to the blood volume fraction and the tracer concentration of reversible compartments. An alternative graphical model for reversible tracers is the Logan plot (69) given by Equation 4:

(4) $$\frac{\int_{0}^{t}C_{\mathrm{PET}}(\tau )d\tau }{C_{\mathrm{PET}}(t)}=K\frac{\int_{0}^{t}C_{P}(\tau )d\tau }{C_{\mathrm{PET}}(t)}+b$$

which requires the integral of $C_{\mathrm{PET}}(t)$ starting from the time of injection in contrast to the Patlak model, which only requires $C_{\mathrm{PET}}(t)$ at time points after steady-state equilibrium between plasma and the non-displaceable compartment. For reversible tracers, the slope *K* of the Logan plot is related to $V_{T}$.

**Figure 3:**
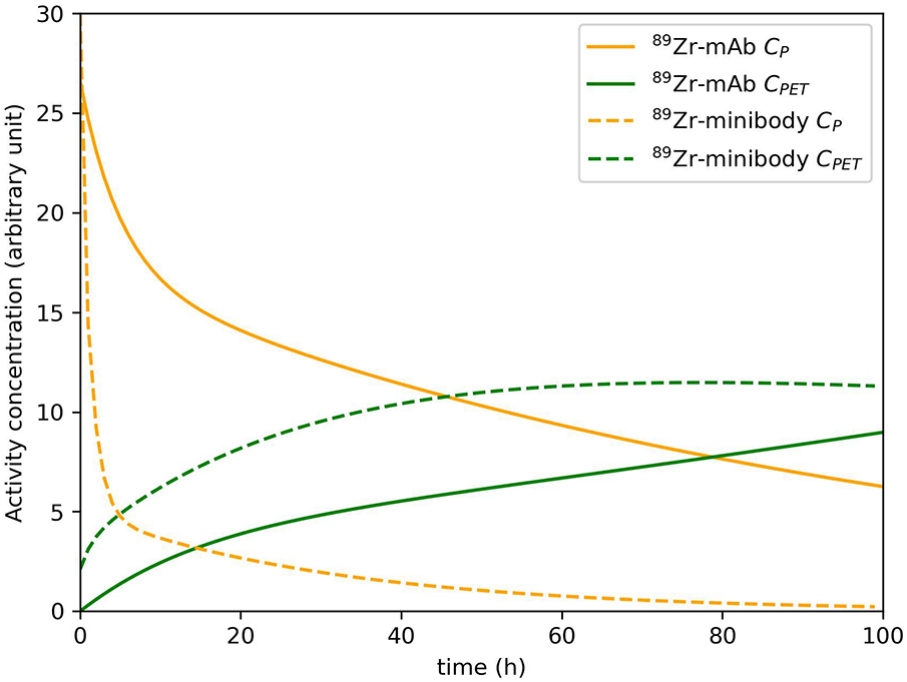
Example plasma concentration $C_{P}$ and tissue concentration $C_{PET}$ for a ^89^Zr-labeled mAb and a ^89^Zr-labeled minibody. These (typical) curves were simulated based on the work of Huisman et al. (71) (mAb) and Omidvari et al. (17) (minibody). It should be noted that plasma and tissue kinetics are highly dependent on the tracer and the target tissue.

As dynamic PET has predominantly been used in brain PET studies (70), it still is an open question to which extent kinetic modeling of uptake of larger molecules in tumors is feasible. Since the PET signal needs to be measured over time, the slow kinetics of most immunoPET tracers requires multiple imaging time-points on different days to capture their kinetics. Figure 3 shows example plasma and tissue TACs for a ^89^Zr-labeled mAb and a ^89^Zr-labeled minibody to illustrate the slow kinetics of these tracer types. In section 4, the choice of imaging time points and other challenging aspects of capturing and analyzing these slow kinetics are discussed further.

## 3 Imaging methodology of immunoPET studies

### 3.1 Semi-quantitative immunoPET studies in humans

Most immunoPET studies have been conducted in a semi-quantitative manner. Depending on the objectives of the study, various study designs have been applied with the main goals of proving safety, determining biodistribution, dosimetry and tumor targeting. Sometimes, biopsies were included in the studies to compare tumor uptake with target expression in biopsies. In addition, a subset of studies attempted to predict response to mAb therapy based on a baseline immunoPET/CT scan or performed a second scan after initiation of therapy. In addition to standard immunotherapy, immunoPET was also investigated regarding applications for RIT and ADCs, where imaging is used for dosimetry calculations or response prediction. An overview of all included studies with semi-quantitative methodologies is provided in Supplementary Table S1 and S2. Exemplary studies are discussed here with respect to the different aspects of immunoPET methodology.

#### 3.1.1 Intact mAbs labeled with ^89^Zr

The majority of published immunoPET studies comprised phase 1 trials of ^89^Zr-labeled intact mAbs, often consisting of two parts. In the first part, the dose escalation phase, different amounts of cold antibody dose were added to the radiotracer and imaging was performed at multiple time-points p.i. In the second part, the dose expansion phase, additional patients were included and usually scans were performed using the optimal dose and imaging time point as determined in the previous part, optimized for visualization of the target, e.g., tumor lesions. Especially the first part of these studies could provide information on how antibody dose affects pharmacokinetics and how tumor uptake changes over time. However, for tumor quantification, only tumor SUVs obtained at later time points were reported predominantly without longitudinal analysis or modeling that takes into account plasma and tissue TACs.

##### 3.1.1.1 The role of cold mAb dose

Often, the radiotracer dose was accompanied by a cold protein dose with the aim of filling a possible antigen-sink and improving lesion visualization. For instance, Dijkers et al. (72) investigated ^89^Zr-trastuzumab in patients with HER2-positive metastatic breast cancer, imaging between D_1_ and D_7_ p.i. using 37 MBq ^89^Zr-trastuzumab. For optimal visualization of tumor lesions, treatment-naive patients required a dose of 50 mg, whereas a dose of 10 mg was sufficient for patients on trastuzumab treatment. The authors pointed out that when only 10 mg is administered to treatment-naive patients, tracer clearance is too fast to allow sufficient accumulation in the tumor. This need for a relatively high dose is rather specific for trastuzumab and is often lower for other mAb tracers.

Another example is the study by Ulaner et al. (73), who investigated CD38-targeted immunoPET of multiple myeloma using ^89^Zr-DFO-daratumumab with antibody doses of 3, 20 and 50 mg. The authors concluded that 20 and 50 mg were similarly effective for imaging, while using 3 mg resulted in high uptake in spleen and liver but not in lesions. The authors discussed that 3 mg may not have been sufficient to saturate nonspecific binding in the liver.

On the other hand, giving a too high cold protein dose may lead to competitive binding between the cold molecule and the tracer and even saturation of the target antigen by unlabeled mAb (72, 74). For instance, Niemeijer et al. (75) investigated ^89^Zr-pembrolizumab in patients with advanced non-small-cell lung cancer (NSCLC). Two imaging series per patient were performed with two injections of 37 MBq ^89^Zr-pembrolizumab (2 mg), one without and one with a pre-dose of 200 mg unlabeled pembrolizumab. The authors observed slower plasma clearance and lower activity concentration in the spleen for the second scan series, using the cold pre-dose, suggesting the spleen functions as a sink. Interestingly, the authors observed that not all tumor lesions identified in part 1, could be assessed in part 2 (19 lesions compared to 10 lesions) and speculated that the pre-dose could have occupied most available PD-1 receptors causing a loss of PET signal.

To summarize, whether and how much cold protein dose should be added varies greatly between different tracers as reported by several more studies (76–80), making a protein dose finding phase necessary. Multiple studies reported potential blocking of tumor binding sites with a too high cold dose, which also might be a concern for performing an immunoPET scan under treatment with high therapeutic doses. In addition, the optimal dose seems to also depend on the individual patient. In a letter to the editor, Oude Munnink et al. (81) reported that trastuzumab pharmacokinetics may be affected by the extent of HER2-positive tumor load, with increased clearance for high tumor loads. They described the case of one patient with extensive tumor load where 50 mg ^89^Zr-trastuzumab resulted in low blood pool levels 2 days p.i., as well as hardly visible bone metastases, whereas a second ^89^Zr-trastuzumab scan shortly after first administrating trastuzumab treatment (220 mg) resulted in a higher blood pool level, less liver uptake, and more uptake in other tumor lesions.

##### 3.1.1.2 Imaging time points

Several phase 1 ^89^Zr-immunoPET studies have determined the optimal imaging time point by performing 2–5 scans between 0.5 h to 10 days p.i., most commonly 3–4 scans between 1 h and 5 days p.i. For instance, the study by Dijkers et al. (72) with 37 MBq ^89^Zr-trastuzumab concluded that scans on D_4_ had an optimal compromise between increased tumor uptake/decreased background uptake and image noise due to too low counting statistics at D_6_ and D_7_. However, this study was performed on an earlier generation PET system (Siemens Exact HR+).

Since the uptake of ^89^Zr-mAbs is considered irreversible, it is generally expected to increase over time if tracer is still present in the blood pool, and therefore a further delayed imaging time point would be beneficial for visualization and TBR. However, how late imaging can be performed is limited by the counting statistics, which depend on the scan time point, the injected activity, the scan duration, and the sensitivity of the PET scanner. Studies conducted in Europe usually applied 37 MBq radioactivity to limit the radiation dose. For instance, ^89^Zr-trastuzumab delivers an effective dose of approximately 0.47 mSv/MBq (82), resulting in about 17 mSv for 37 MBq ^89^Zr-trastuzumab. Laforest et al. (82) performed imaging with a higher activity of 62 MBq and recommended imaging at D_6_, in contrast to D_4_ as Dijkers et al. recommended (72).

Another example is the study by Pandit-Taskar et al. (83), who investigated ^89^Zr-huJ591 targeting PSMA using even 185 MBq together with 25 mg protein dose in metastatic prostate cancer patients with multiple scans up to 8 days p.i. Lesion SUV_LBM−max_ values were highest at the latest imaging time point. Subsequently, the same group performed a phase I/II study for validation of ^89^Zr-huJ591 (84) in fifty patients. Here, the best target-to-background ratios were achieved using the latest imaging time points between D_6_ and D_8_ p.i. Therefore, for ^89^Zr-labeled mAbs, it can be concluded that SUV uptake and TBRs increase with later imaging time points. Further examples for studies including different imaging time points with ^89^Zr-labeled mAbs can be found in (85–90). It should be noted that the sensitivity of LAFOV PET/CT scanners allows for a further delay of the imaging time-point with acceptable image quality (91).

##### 3.1.1.3 Correlation to biopsies and treatment response

Important questions include whether immunoPET can distinguish between tissues with and without target expression and whether it can distinguish between different levels of target expression. For example, high HER2 expression was correlated with a better response to trastuzumab-based therapies (92) and therefore immunoPET may aid clinical decision-making (93). However, most immunoPET studies only included a small sample size, often between 6 and 12 patients, rarely up to 50–90 patients, making it difficult to find statistically significant correlations between imaging findings and target expression or response to therapy (75). For example, Bahce et al. (94) performed a pilot study using ^89^Zr-bevacizumab in 7 non-small cell lung cancer patients using imaging before start of carboplatin-paclitaxel-bevacizumab chemotherapy. A positive trend but no significant correlation between lesion SUV_peak_ and overall survival (OS) or progression-free survival (PFS) was observed. Similarly, in a study by Smit et al. (76), who investigated ^89^Zr-durvalumab in 13 NSCLC patients, tumor uptake was higher in patients with treatment response or stable disease compared with patients with disease progression, but these results were not statistically significant (*P* = 0.06).

A few studies could demonstrate a significant correlation between semi-quantitative PET metrics like SUV_max_ or SUV_peak_ and target expression assessed by biopsies. For instance, a study by Dehdashti et al. (95), considering 46 breast cancer patients, suggested that ^89^Zr-trastuzumab SUV_max_ could differentiate between HER2-positive and HER2-negative tumor lesions. When excluding hepatic lesions, tumor SUV_max_ was significantly higher in HER2-positive compared with HER2-negative patients (*P* = 0.003), potentially enabling a SUV_max_ cutoff. However, when considering all tumor lesions, HER2-positive patients did not show significantly higher SUV_max_ (*P* = 0.06), suggesting that semi-quantitative PET metrics for lesions located in the liver may not reflect HER2 status of those lesions. Niemeijer et al. (96) applied ^89^Zr-nivolumab in a study including 13 non-small-cell lung cancer patients and could demonstrate a significant correlation between ^89^Zr-nivolumab lesion SUV_peak_ and both PD-1 positive tumor-infiltrating immune cells and response to nivolumab treatment at a lesion-level, when excluding lesions with a diameter less than 20 mm to avoid PVE. In the same study, a smaller ^18^F-labeled molecule targeting anti-PD-L1 (Adnectin) was investigated with similar promising results and the advantage of same-day imaging. Kok et al. (97) conducted a study on ^89^Zr-pembrolizumab with the goal of assessing clinical response to PD-1 blockade in 18 patients with advanced metastatic melanoma or NSCLC. Tumor uptake correlated with response (*P* = 0.014), PFS (*P* = 0.0025) and OS (*P* = 0.026). However, two tumor biopsies taken after the last PET scan on D7 were negative for PD-1 in immunohistochemistry (IHC), but showed high uptake on PET (SUV_max_ 17.0 and 21.0).

The previous examples as well as further studies (78, 98–100) found discrepancies between PET uptake and biopsy results, suggesting several reasons for these differences, including possible errors in pathology sampling and processing, lesion heterogeneity, but also nonspecific ^89^Zr-mAb uptake and free ^89^Zr uptake in bone lesions. In addition, PET-negative lesions may have other characteristics than PET-positive lesions leading to lack of tracer permeability and retention. It should be noted that, recently, possible evidence for non-specific, irreversible ^89^Zr-mAb uptake in biopsy proven target-negative tumors was presented (101).

Because of the aforementioned limitations of biopsies, correlation of immunoPET markers with treatment response are of particular interest. Bensch et al. (102) explored ^89^Zr-atezolizumab for response prediction of PD-L1 blockade therapy. The authors compared PD-L1 expression assessed by IHC and RNA sequencing of pre-treatment biopsies with immunoPET uptake. Patients with complete response had a 235% higher ^89^Zr-atezolizumab SUV_max_ than patients who immediately progressed. The geometric mean SUV_max_ per patient was strongly related to PFS and OS. At lesion level, uptake was also related to change in size during treatment. PD-L1 IHC or RNA-sequencing-based predictive biomarkers of pre-treatment biopsies did not correlate with PFS and OS.

On the other hand, mixed results were found by Gaykema et al. (103) for ^89^Zr-trastuzumab and ^89^Zr-bevacizumab to evaluate the effects of the HSP90 inhibitor NVP-AUY922 in patients with advanced HER2-positive (trastuzumab) or ER-positive (bevacizumab) breast cancer. Each patient underwent a PET scan on D_2_ and D_4_, once at baseline and once at day 15 of therapy cycle 1. SUV_max_ was reported and the immunoPET scans were compared to response on CT and ^18^F-FDG PET after 8 weeks. A moderate correlation was found between the mean decrease in SUV_max_ on ^89^Zr-trastuzumab PET scans after 3 weeks of treatment, and the change in tumor lesion size on CT after 8 weeks of treatment compared with baseline. However, for ^89^Zr-bevacizumab liver lesions could not be visualized in most cases, due to high physiological liver uptake. Furthermore, SUV_max_ on D_4_ was unaffected during treatment, indicating that no correlation between changes in PET uptake and lesion size on CT was found. Another example is the study by van Helden et al. (104) reporting that tumor uptake of ^89^Zr-cetuximab failed to predict treatment benefit in patients with RAS wild-type mCRC receiving cetuximab monotherapy. Interestingly, this study included a ^15^O-H_2_O PET/CT scan to determine tumor perfusion, which was positively correlated with ^89^Zr-cetuximab SUV_mean_ on a lesion level.

Further studies, including correlations of ^89^Zr-mAb PET uptake with biopsies or treatment response, can be found in (12, 75, 102, 105–112) as well as in Supplementary Table S1. Other ^89^Zr-mAb immunoPET studies are ongoing such as the IMPACT-MBC study (NCT01957332), which also includes biopsies and is worth mentioning because of the larger patient population of 100 patients.

#### 3.1.2 Intact IgGs labeled with ^124^I

Guo et al. (113) reported on ^124^I-trastuzumab in gastric cancer patients. Imaging was performed in 6 patients between 1 h and 96 h p.i. with, on average, 74 MBq ^124^I-trastuzumab with a co-injection of either 5 mg or 10 mg trastuzumab. The optimal imaging time point was at 24 h p.i. showing the highest lesion SUV_max_. At 48 h p.i., image quality was poorer due to low count rates. The authors suggested that, compared with ^89^Zr and ^64^Cu, ^124^I-trastuzumab may achieve higher imaging contrast because of lower nonspecific uptake and better TBR for soft tissues, especially in the liver. In contrast to ^89^Zr and ^64^Cu, ^124^I is not residualizing and thus, after degradation, the radionuclide may diffuse freely from the tissues, resulting in the loss of specific signal in target tissues and increased background signal over time, leading to earlier optimal imaging time points p.i. with respect to the residualizing radiometals.

Carrasquillo et al. (114) investigated an ^124^I-labeled mAb, huA33, in 25 patients with colorectal cancer. Patients received 343 MBq/10 mg of the mAb and imaging was performed ranging from 45 min to 8.9 days p.i. SUV_max_ of the latest imaging time point was compared with an intermediate time point on D_2_. In three tumors, no change or an increase in uptake was seen, but 14 tumors showed a decrease in uptake. This is in contrast to ^89^Zr-immunoPET where ^89^Zr is remains in the cell after degradation, leading to accumulation of the signal over time. In a follow-up study, Donoghue et al. (115) performed only one scan approximately 1 week later, followed by a detailed assay of surgically removed tissue. The spatial distribution of ^124^I-huA33 conformed to that of the A33 antigen, with a linear relationship between the amount of bound antibody and antigen concentration. In a later publication, Zanzonico et al. (116) applied compartmental modeling to ^124^I-huA33 antibody imaging, which will be described in section 3.2.

Carrasquillo et al. (117) also studied ^124^I-codrituzumab imaging in 13 patients with hepatocellular carcinoma using 185 MBq, 10 mg tracer dose. Seven of the patients not only received a scan at baseline but also a second scan after undergoing sorafenib/immunotherapy with 2.5 or 5 mg/kg of cold codrituzumab dose. They chose ^124^I instead of ^89^Zr, primarily because of the concern that ^89^Zr would result in higher liver background uptake, due to the slow internalization rate of the target of codrituzumab (Glypican-3), and lack of access to ^89^Zr. In most patients, tumor uptake concentration peaked at 24 h and slowly decreased afterward, which the authors considered to be uncommon for intact IgG and may be related to the high vascularity of hepatocellular carcinoma or specific for the Glypican-3 target.

#### 3.1.3 Intact IgGs labeled with ^64^Cu

Mortimer et al. (118) investigated ^64^Cu-DOTA-trastuzumab imaging in treatment-naive HER2-positive metastatic breast cancer patients. They applied 50 mg protein dose and 364–512 MBq ^64^Cu-DOTA-trastuzumab, imaging on D_1_ and D_2_ p.i. They found a detection sensitivity of 77% and 89% for D_1_ and D_2_ compared with 93% for ^18^F-FDG. In a later study, Mortimer et al. (119) investigated tumor uptake of this tracer in patients with both HER2-positive and HER2-negative disease based on IHC of tumor biopsies. Median SUV_max_ for D_1_ and D_2_ was 6.6 and 6.8 for HER2 positive patients, respectively, and 3.7 and 4.3 for HER2-negative patients. The distributions of SUV_max_ overlapped between the 2 groups and interpatient variability was greater for HER2-positive than for HER2-negative disease. Jarret et al. (120) demonstrated with two patients a mathematical model combining ^64^Cu-DOTA-trastuzumab PET/CT with MRI information to predict therapy response at the time of surgery after neoadjuvant therapy with a combination of chemotherapy, trastuzumab and pertuzumab in HER2-positive breast cancer. ^64^Cu-DOTA-trastuzumab was also applied for visualizing HER2-positive brain metastasis by Kurihara et al. (121).

Carrasquillo et al. (122) performed a study with ^64^Cu-trastuzumab (296–370 MBq/5 mg) with 11 patients on trastuzumab treatment of which 8 patients underwent a repeat scan. Since ^64^Cu has a shorter half-life than ^89^Zr, they limited imaging to D_1_ (24 h) p.i. The repeated scans showed acceptable reproducibility of biodistribution and pharmacokinetic clearance. However, only in 2 of the 11 patients tumors were visualized. The authors discussed that this could be due to the chronic high-dose treatment of trastuzumab, which was expected to compete with the small dose of ^64^Cu-trastuzumab.

Lockhart et al. (123) performed a phase 1 evaluation of ^64^Cu-DOTA-patritumab (targeting HER3) in patients with advanced solid tumors in two different cohorts. In a dosimetry cohort, patients were scanned at 3 h, D_1_ and D_2_ p.i. with the tracer (300–555 MBq/0.2 mg), which revealed D_1_ as the optimal imaging time point. In the second cohort, patients were administered with the tracer twice (one week apart) followed by a PET/CT at D_1_ p.i. For the second scan, each patient who had detectable tumor uptake on the baseline scan received 8.0 mg/kg unlabeled patritumab (3 h before radiotracer injection). The average tumor SUV_max_ of the three patients with detectable uptake, at baseline and after unlabeled patritumab dosing was 2.90 ± 0.72 and 4.01 ± 1.32, respectively. The corresponding average TBR were 1.00 ± 0.32 and 0.57 ± 0.17, respectively. Receptor occupancy was determined as $(VT_{d-}-VT_{d+})/VT_{d-}$, where VT was denoted as the volume of distribution defined as the ratio of tumor activity to blood pool activity for each lesion and *d*- and *d*+ were baseline and high cold dose scans. This resulted in a receptor occupancy of 42.1 ± 3.9%. The authors acknowledge that the simple analysis used did not account for potential complexities related to rate of blood clearance, changes in organ or tissue uptake, or receptor internalization. They also mentioned that the increased in SUV_max_ with cold dose was not expected from preclinical studies, and attributed this to the 3 h time delay between cold dose administration and tracer injection and the higher circulating levels of the tracer dose in the blood after pre-dosing.

Krishnan et al. (124) performed a first-in-human imaging study using ^64^Cu-daratumumab in 12 myeloma patients using 555 MBq/5 mg tracer with additional varying amounts of cold doses (0, 10, 45 or 95 mg). Scans were performed on D_0_, D_1_ and D_2_ p.i. Here, 45 mg was found to be favorable in terms of removing background signal without saturating target sites.

The articles reviewed in this section mentioned the advantage of ^64^Cu over ^89^Zr as being able to inject more activity because of the lower radiation dose, especially with radiotracer uptake in bone marrow. ^89^Zr on the other hand would be advantageous for tracers where the disease related signal is difficult to differentiate because of background tissue uptake, since it enables to image at delayed time points with higher (irreversible) tracer uptake combined with more clearance from the background tissues. Indeed, a few studies found lack of sufficient visualization of target regions with ^64^Cu-immunoPET imaging, especially in the liver (125), and discussed using ^124^I or ^89^Zr instead (125, 126).

#### 3.1.4 Smaller mAb based constructs

Pandit-Taskar et al. (127) performed a first in-human imaging study with ^89^Zr-Df-IAB2M, an anti-PSMA-minibody, in metastatic prostate cancer patients. In a phase I dose-escalation study, patients received 185 MBq of ^89^Zr-IAB2m and cold Df-IAB2m at total mass doses of 10, 20 and 50 mg. Each patient underwent 4 PET/CT scans, including one on the day of injection, and additional scans at D_1_, D_2/3_ and at D_4/5_ p.i. With 50 mg, SUV_max_ in lesions was significantly lower, and with 10 mg the highest uptake was seen. More lesions were detectable at 48 h compared with 24 h p.i. Delayed imaging at 72–120 h p.i. showed additional lesions in a few patients. Lesion uptake and TBR increased in most tumor lesions throughout all imaging time points. However, the authors concluded that imaging 48 h p.i. provides sufficient visualization.

Pandit-Taskar et al. (128) and Farwell et al. (129) reported on the first-in-human imaging with ^89^Zr-Df-IAB22M2C, an anti-CD8 minibody in patients with solid malignancies. Patients with different types of cancer received 111 MBq and variable cold protein dose ranging from 0.2 mg up to 10 mg in part 1 of the trial, and 0.5 mg or 1.5 mg in part 2 of the trial because of improved visualization. Each patient had 4–5 PET scans at 2–4 h, 6–8 h, D_1_, D_2_ and D_4_-D_6_ p.i. In general, the results showed that cold minibody mass dose influences the biodistribution and targeting of CD8+ T-cell-rich tissues, with uptake being inversely affected by the cold mass. Multiple-time-point imaging showed variable trends with stable uptake in CD8+ tissues and clearance of blood-pool and background activity. However, nodal uptake varied in time with minibody mass change revealing more lymph nodes at early time points (6–24 h p.i.) at lower masses compared with higher masses (5 or 10 mg), suggesting a strong effect of competitive binding. The variable uptake was also discussed to potentially be caused by the treatment profile or the variable presence of CD8+ T-cells. Tumor uptake quantified as SUV_max,LBM_ and SUV_peak,LBM_ was maximal at 24–48 h p.i. Uptake in CD8-rich tissues was saturable with lower uptake in the spleen and bone marrow in the 1.5 mg compared with the 0.5 mg cohort. However, no differences were seen in lymph node uptake, which the authors hypothesized to be due to higher blood flow and availability of target sites in the spleen and bone marrow compared with lymph nodes. The authors acknowledge the limitations of a heterogeneous small patient population with different tumor types, tumor burden and treatment history, as well as lack of correlative biopsy data. A phase 2 trial testing the diagnostic and predictive performance is ongoing (NCT03802123). Schwenck et al. (130) investigated the same tracer, ^89^Zr-Df-IAB22M2C, in 8 patients with metastasized cancers undergoing immune checkpoint inhibitor therapy using PET/MRI at 24 h p.i. In this small cohort, uptake in metastatic tumor lesions was unable to predict treatment response. Interestingly, they discussed that cancer progression was associated with a relatively low spleen-to-liver uptake ratio and also that dual tracer approaches might be required for the identification of efficient CD8+ T-cell function, which may potentially enable the prediction of therapy response.

Moek et al. (131) studied the ^89^Zr-labeled (37 MBq) bispecific T-cell engager AMG 211 directed against carcinoembryonic antigen (CEA) and CD3 on T-cells. The tracer was administered alone or with cold AMG 211 in patients with proven gastrointestinal adenocarcinomas. Imaging was performed before treatment and after the end of the second AMG 211 treatment period. Prior to treatment the optimal dose was 2 mg (0.2 mg tracer plus 1.8 mg cold dose) with a tracer serum half-life of 3.3 h. According to the authors the development of bispecific antibodies is more challenging due to the two arms that differ in binding affinity for targets. Regarding PET image analysis, bi- or multi-specific tracers are an additional challenge since the PET signal cannot differentiate between the signals from individual targets. The authors observed increased uptake even after tracer washout of the blood. High intra- and interpatient heterogeneity in ^89^Zr-AMG 211 tumor accumulation was seen before AMG 211 treatment that might be due to target expression as well as delivery by tumor vasculature, and tissue permeability. In general, smaller antibody constructs result in faster tracer kinetics that lead to earlier imaging time points with respect to the more commonly used imaging time points for intact antibodies. The treatment group showed an altered biodistribution leading to high and sustained ^89^Zr-AMG 211 presence in the blood pool and absence of tumor lesion visualization, which may indicate tumor target saturation.

Kist de Ruijter et al. (132) performed CD8+ T-cell imaging before and during immunotherapy with atezolizumab using the one-armed antibody tracer ^89^ZED88082A in patients with solid tumors. In the dose-finding part A of the study, patients received the tracer consisting of 37 MBq and 1.2–1.5 mg with an additional unlabeled CED88004S until a total protein dose of 4 mg or 10 mg. PET scans were performed at 1 h, D_2_, D_4_ and D_7_ p.i. followed by a biopsy after the last PET scan. After the baseline PET scans, patients received 1,200 mg atezolizumab every three weeks. The average tumor SUV_max_ was calculated as a geometric mean of tumor uptake values per patient. Imaging with 10 mg cold antibody dose on D_2_ p.i. was considered optimal. Higher SUV_max_ was associated with longer OS. ImmunoPET suggested large heterogeneity in CD8+ T-cell distribution and pharmacodynamics within and among patients.

Thorneloe et al. (133) investigated albumin-binding domain antibodies (AlbudAbs™) as a method to extend the half-life and alter the distribution of biological and small molecule therapeutics in tissues. AlbudAbs bind albumin with high affinity and can be fused or conjugated to therapeutic agents. ^89^Zr-immunoPET was used to study the distribution properties of an AlbudAb with 4 scan time points up to 7 days p.i. using, on average, 14 MBq in healthy males with a radiation exposure below 10 mSv. Due to the size of the AlbudAb-albumin complex, the extravasation was similar to that of antibodies. The authors compared for several organs the observed TPR with a theoretical expected range that was calculated based on (134) to be between a minimum (if tracer concentration would be confined to vasculature only) and a maximum (if tracer concentration in organ interstitial space would be equal to plasma). For example, in lungs, liver, and spleen the highest expected TPR was reached quickly suggesting a rapid distribution, whereas in brain, the TPR stayed at the theoretical minimum suggesting the tracer was confined to the brain vasculature.

Heuveling et al. (135) reported on a pilot immunoPET study using an ^124^I-labeled F16SIP minibody. ^124^I was used because this minibody binds to a non-internalizing extracellular matrix target. Imaging was performed at 30 min and 24 h p.i. in 4 patients following administration of 74 MBq. The study demonstrated biodistribution and tumor targeting, warranting further clinical investigation of this molecule.

A study by Laforest et al. (136) investigated the ^124^I-labeled F(ab')_2_ antibody fragment PGN650 targeting phosphatidylserine in the tumor microenvironment. They applied 140 MBq tracer dose and imaging time points at 1 h, 3 h, 24 h, and 48 h with only mildly increased uptake in tumors, which was highest at the latest imaging time point.

Scott et al. (137) investigated an ^124^I-labeled pegylated diabody PEG-AVP0458 in patients with tumor-associated glycoprotein 72 positive cancers in xenografts and in 5 patients. Imaging in patients was performed using 111–185 MBq at a protein dose level of 1.0 or 10 mg/m^2^ on D_0_, D_1_, D_2/3_, D_4/5_, and D_6/7_ p.i. Imaging showed rapid and highly specific targeting of tumor lesions and minimal normal organ uptake leading to high tumor-to-background ratios. This behavior of the pegylated diabody (138) was discussed to be favorable compared with other mAb based fragments.

Liu et al. (139) performed a comparison of two HER2-targeting ^18^F-labeled affibodies in mice and breast cancer patients. Patients were scanned on the uEXPLORER LAFOV PET scanner using 231 MBq tracer with a co-injection of 1 mg cold HER2 affibody at scan time points of 2 h and 4 h p.i. One patient also underwent dynamic PET acquisition from 0 to 45 min p.i., however no kinetic modeling was performed. Besides other aspects, they discussed that the half-life of ^18^F suits affibodies well and enables imaging at 4 h p.i. with higher SUV_max_ and improved contrast compared with the 2 h time point.

Zhou et al. (140) performed HER2 imaging in gastric cancer patients with HER2-positive and HER2-negative primary lesions using a ^68^Ga-labeled affibody. Imaging was performed at 1 h, 2 h, and 3 h p.i. using 3.7 MBq/kg ^68^Ga-HER2 affibody with 0.5 mg cold affibody to help reduce nonspecific organ uptake. SUV_max_ was used to quantify lesions with optimal lesion visualization contrast at 2 h p.i. Imaging at 4 h p.i. was expected to be insufficient due to poor count statistics and rapid clearance of this tracer. The HER2-positive group had significantly higher uptake than the HER2-negative group and a possible SUV_max_ cutoff was discussed. However, overall, high variability in SUV_max_ was found, suggesting heterogeneity in HER2 expression with organ-dependent differences in HER2-positive patients. Patients with high uptake showed longer PFS. Interestingly, they discussed that ongoing HER2 therapy did not influence ^68^Ga-affibody imaging because the affibody would mainly bind to domain III of the extracellular portion of HER2, while trastuzumab and pertuzumab bind to domains IV and II, respectively. The latter would enable monitoring and re-evaluation of the HER2 status during therapy. On the other hand, however, uptake may not directly translate to uptake of anti-HER2 mAb treatment, which may affect response prediction. A study of the same tracer in breast cancer patients with imaging at 2 h p.i. was performed by Miao et al. (141) and one of the first reports on a different ^68^Ga-labeled affibody targeting HER2 can be found in (142).

Beylergil et al. (143) performed a pilot study on ^68^Ga-DOTA-F(ab')_2_-trastuzumab in patients with breast cancer. 16 patients were imaged at average time points of 1.1, 1.8 and 2.7 h p.i. using 236 MBq/5 mg tracer on average. Semi-quantitative analysis showed minimal or no tumor uptake in most cases, which was attributed to patient selection, immunoreactivity and suboptimal antibody mass.

Sandström et al. (144) investigated the anti-HER2 affibody ^68^Ga-ABY-025 in breast cancer patients, including a dynamic 45 min acquisition followed by three static scans at 1, 2, and 4 h p.i. using on average 215 MBq. TACs were acquired and used for radiation dosimetry, which was around 6.0 mSv for a low- (78 μg) and 5.6 mSv for a high cold dose (427 μg). Higher detection rate and image contrast were favourable with a higher cold dose and the optimal imaging time point was set at 2 h p.i. In a consequent publication, Sörensen et al. (145) performed a successful test-retest study with a second ^68^Ga-ABY-025 PET/CT scan one week later finding an intraclass correlation of *r* = 0.996. In addition, biopsies from 16 metastases in 12 patients were collected. Imaging at 4 h p.i. with high cold dose (427 μg) correlated well with biopsy HER2-scores (*r* = 0.91, *p* < 0.001) and uptake was five times higher in HER2-positive than in HER2-negative lesions with no overlap (*P* = 0.005).

Wang et al. (146) performed a pilot study for the nanobody ^68^Ga-NODAGA-SNA006 targeting CD8+ T-cells in mice, monkeys, and in three lung cancer patients. One monkey was scanned with 25 μg/kg and the other with a blocking dose of 150 μg/kg. Imaging was performed at 0.25, 0.5, 1, 1.5, 2, and 4 h p.i. Patients received on average 163.8 MBq, and 100 μg or 800 μg mass dose, and underwent three PET/CT scans at 15–30 min, 60–90 min, and 120 min p.i. Fast washout (*t*_1/2_ < 20 min) was observed in the three patients. A preliminary linear relationship was found between uptake and CD8 expression with IHC. On the higher cold dose, liver uptake was decreased. The results in the primates showed highest uptake in the spleen, which was reduced by more than 90% with the higher cold dose. The uptake in tumors plateaued within 15 min p.i. This is in contrast to the previously mentioned anti CD8 + minibody ^89^Zr-IAB22M2C, which showed highest uptake at D_1_ or D_2_ p.i.

In a phase 1 study, Gondry et al. (147) evaluated the use of ^68^Ga-Anti-CD206-sdAb for assessing protumorigenic macrophage presence in solid tumors. A total of seven patients were imaged with on average 181 MBq/80 μg of the tracer at 11 min, 90 min and 150 min p.i. The blood clearance was fast with less than 20% of the activity remaining after 80 min. Preliminary data showed higher uptake in the three patients that progressed compared with three without progression, suggesting further investigation of this tracer in phase II clinical trials (NCT04168528, NCT04758650).

Keyaerts et al. (148) investigated a ^68^Ga-HER2-nanobody (trade name of Ablynx) in a phase I study to assess HER2 expression in breast cancer patients. PET/CT scans for dosimetry assessment were obtained at 10, 60, and 90 min p.i. using on average 107 MBq tracer. Fast blood clearance was observed with only 10% of activity remaining in the blood at 1 h p.i. Tumor uptake was evaluated using SUV_mean_ and a wide range was observed, but no histopathologic correlation was performed in this study. The authors highlighted the advantage of nanobody binding to a different epitope as the therapeutic agent, as it may be less affected by any circulating therapeutic compounds. On the other hand, it should be noted that the potential for predicting response to anti-HER2 therapy still needs to be investigated, especially given this difference between tracer and therapeutic compound.

Recently, Li et al. (149) published an article about a CEA-targeted ^68^Ga-nanobody in both rodents and patients. Patients received on average 151 MBq and underwent PET/CT scans at 1 h and 2 h p.i., whereas the first patients also underwent a dynamic PET scan from 0 to 40 min p.i. Fast blood clearance and low background uptake were observed and the colorectal carcinoma lesions could be visualized as early as 30 min p.i.

#### 3.1.5 ImmunoPET for radioimmunotherapy or immunoconjugates

Rizvi et al. (150) applied ^89^Zr-ibritumomab tiuxetan imaging for radiation dosimetry and scouting of ^90^Y-ibritumomab tiuxetan therapy in patients with relapsed B-cell non-Hodgkin's lymphoma. Imaging was performed in 7 patients after injection of 70 MBq ^89^Zr-ibritumomab at 1, 72 and 144 h p.i. and again 2 weeks later, this time with co-injection of 15 MBq/kg or 30 MBq/kg ^90^Y-ibritumomab tiuxetan. Absorbed doses to healthy organs and tumors were calculated. The correlation between predicted pre-therapy and on-therapy organ absorbed doses was high (Pearson *r* = 0.97). Correlation between pre-therapy and on-therapy tumor absorbed doses was lower (*r* = 0.75) but not significantly different.

Muylle et al. (74) studied tumor targeting and radiation dose of RIT with ^90^Y-rituximab in CD20+ B-cell lymphoma after ^89^Zr-rituximab immunoPET and the impact of preloading with unlabeled rituximab. Five patients underwent three study phases: a ^89^Zr-rituximab scan without a cold preload, a ^89^Zr-rituximab scan with 250 g/m^2^ preload, and ^89^Y-rituximab therapeutic phase including unlabeled rituximab. Without the cold rituximab preload, a much higher dose to the spleen was observed, but also higher tumor uptake in patients with B-cell depletion. The authors concluded that administration of the standard preload impairs tumor targeting in patients that were previously treated with rituximab. Other studies exist that show immunoPET imaging may help to predict absorbed doses in RIT by using diagnostic and therapeutic nuclide pairs like ^89^Zr and ^177^Lu (106) or ^124^I and ^131^I (151, 152). It is worth mentioning that ^89^Zr-labeled immunoPET imaging is also being investigated in preclinical research prior to targeted alpha therapy (153).

Several studies investigated the potential to use immunoPET imaging prior to therapy with an immunoconjugate like an immunocytokine or ADCs. Van Brummelen et al. (154) applied ^89^Zr-immunoPET imaging for targeted immunocytokine cergutuzumab amunaleukin (CEA-IL2v) in different types of CEA-positive and CEA-negative cancer patients. Different treatment dose cohorts were used with imaging time points ranging from D_1_ to D_9_, analyzed using %ID (injected dose)/mL_peak_ values. Using imaging data from this study, a PK/PD mathematical model was created (155) with regard to dosing and scheduling for early dose-finding clinical studies.

In a study including 90 patients, Mileva et al. (156) performed ^89^Zr-immunoPET and FDG PET before T-DM1 therapy in advanced HER2-positive breast cancer patients. Lesions were visually classified as HER2-positive and HER2-negative. The authors concluded that ^89^Zr-immunoPET, alone or in combination with FDG PET, can successfully identify breast cancer lesions and patients with a low probability of clinical benefit from T-DM1 therapy.

Smaller mAb-based constructs labeled with other radionuclides can also be used prior to ADC therapy. For instance, Natarajan et al. (157) investigated a ^64^Cu-labeled mAb fragment for measuring CA6 expression in cancer for ADC therapy.

### 3.2 Quantitative immunoPET studies in humans

Only 10 articles on clinical immunoPET went beyond semi-quantitative PET methodology and are reviewed in detail here. An overview of these articles is provided in Supplementary Table S3.

#### 3.2.1 Time-activity curves and Patlak modeling with intact ^89^Zr-mAbs

**Figure 4:**
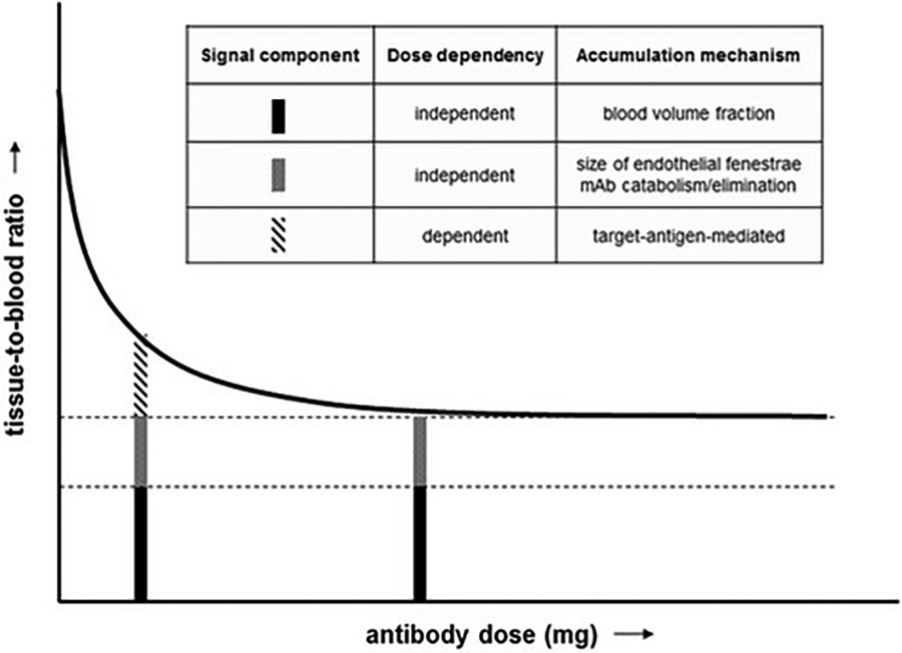
Immuno-PET signal components in a phase 1 dose escalation study. Tissue-to-blood ratio as a function of antibody dose. Figure taken from Jauw et al. (CC BY 4.0 License) (158).

Jauw et al. (158) performed a phase I immunoPET study using a ^89^Zr-labeled anti-CD44 tracer (RG7356) focusing on dose-dependent uptake in normal tissues. The study assumed that target antigen-mediated uptake is dose-dependent, while blood volume fraction, catabolism or elimination are dose-independent signal components (see also Figure 4). The tracer ^89^Zr-anti-CD44 was administered using 37 MBq and 1 mg mass dose, after a variable dose of the unlabeled mAb (0–675 mg). The area under the curve (AUC) of tracer uptake in normal tissues was determined and expressed as tissue-to-blood AUC ratios. In the brain a constant tissue-to-blood AUC ratio was observed for all dose cohorts, indicating RG7356 does not cross the blood-brain barrier, whereas for all other normal tissues the tissue-to-blood AUC ratios decreased with increasing cold mAb dose up to 450 mg, indicating target antigen-mediated specific uptake. A constant ratio was reached at 450 mg suggesting target antigen saturation. In the lowest dose cohorts, no focal tumor uptake was visualized, which the authors attributed to the uptake in normal tissues. Tumor uptake of the antibody was observed in all patients receiving 450 mg or more. Tumor-to-blood AUC ratios were on average 0.46 ± 0.15 for 450 mg and 0.65 ± 0.07 for 675 mg. The authors discussed that to exclude differences in tumor characteristics, a different study design would be more informative, e.g., measuring the same tumor after injection of different antibody doses and performing biopsies after immunoPET.

In another study, Jauw et al. (159) performed a retrospective study with data of 4 different ^89^Zr-mAb tracers: obinutuzumab (anti-CD20) (160), cetuximab (anti-EGFR) (108), huJ951 (anti-PSMA) (83), and trastuzumab (anti-HER2) (85). The authors applied Patlak modeling to estimate the reversible and irreversible contributions to the total measured uptake in kidneys, liver, lung and spleen. They compared the measured $K_{i}$ with literature $K_{i}\;$ values based on physiological components for nonbinding intact IgG1 mAb using the antibody biodistribution coefficients for various tissues that were obtained using PBPK modeling validated with experimental data (161). For ^89^Zr-anti-PSMA, a 4-fold higher $K_{i}$ for the kidneys was observed indicating target engagement. It needs to be noted that perfusion is higher in kidneys than in other organs, which may be a confounding factor for $K_{i}$. Nonspecific uptake accounted for 66%, 34% and 22% of the total signal in the kidneys at D_1_, D_3,_ and D_7_ p.i. The authors concluded that nonspecific uptake of ^89^Zr-labeled mAbs in normal tissues can be quantified using Patlak. However, they also discussed that tumors are more complex than normal tissues, so that nonspecific, reversible uptake in tumors may be more variable between tumors and patients.

Miedema et al. (162) investigated a ^89^Zr-anti-LAG-3 tracer in head and neck, and lung cancer patients. Patients underwent imaging with ^89^Zr-BI854111 (anti-LAG-3) using 4 mg protein mass and 37 MBq tracer dose. PET scans were performed at 2 h, D_4,_ and D_6_ p.i. To investigate target specificity, the authors administered a second tracer dose two weeks later with a pre-dose of 40 or 600 mg unlabeled anti-LAG-3 followed by scans at D_4_ and D_6_. The authors discussed that a relatively low target expression, as described for LAG-3, as well as a small variation in target expression can be overshadowed by temporal differences in plasma availability of the tracer. They acknowledged that one way to address this would be to perform Patlak analysis, however, in contrast to normal organs, baseline $K_{i}$ for tumors lacking target expressions would not be available. Therefore they decided to report only $K_{i}$ values for organ tissues and TPR for lesions. They observed partial saturation of the spleen uptake at 44 mg and full saturation at 604 mg based on Patlak $K_{i}$ values. TPRs were favorable at the 4 mg mass dose and increased over time while adding cold dose decreased TPR. In another study by Miedema et al. (163), this method of comparing $K_{i}$ values with baseline $K_{i}$ values for non-specific organ uptake was applied on data of several other mAb tracers (nivolumab, pembrolizumab, durvalumab, BI 754111, and ipilimumab). For example, all of the tracers mentioned, except ipilimumab, showed $K_{i}$ values for the spleen that were above the determined baseline value indicating target engagement. In addition, decreasing $K_{i}$ values were observed with higher mass doses. This suggests that Patlak analysis in combination with baseline $K_{i}$ values for non-specific organ uptake can detect target engagement in healthy organs.

Menke-van der Houven van Oordt et al. (164) studied an anti-HER3 mAb (GSK2849330) to investigate its biodistribution, tumor uptake, as well as target engagement as function of antibody mass dose. Patients received two administrations of ^89^Zr-anti-HER3; the first imaging time point was performed at baseline after administration of tracer alone (part 1). At the time of the second administration (part 2), two weeks later, dose-dependent inhibition of tracer uptake in tumor tissue was assessed by dose escalation of unlabeled mAb in combination with the tracer. PET scans were performed 2 h p.i. and on D_2_ and D_5_ p.i. Biodistribution and tumor targeting were assessed using SUV, and Patlak analysis was used to calculate the 50% and 90% inhibitory mass doses ($ID_{50}$ and $ID_{90}$) of target-mediated uptake. Pre-dosing with unlabeled mAb reduced tumor uptake rate in a dose-dependent manner. Saturation of tumor uptake was seen at the highest dose (30 mg/kg). Although the study only included six patients and did not cover intermediate doses from 1 mg/kg to 24 mg/kg, an exploratory ID_50_ of 2 mg/kg and ID_90_ of 18 mg/kg was determined by fitting a dose inhibition curve to the *K_i_* ratio of part 2 (pre-dosing) to part 1 (tracer only) of the study. In addition, only two late imaging time-points were used, which allowing linear fitting without goodness of fit assessment. No statistical analysis was performed due to the small number of patients.

Wijngaarden et al. (39) aimed to assess the validity of the semi-quantitative parameters SUV, TPR, and TBR against Patlak K_i_. The study was based on retrospective data of two ^89^Zr-immunoPET studies: ^89^Zr-anti-EGFR (cetuximab) (108) and ^89^Zr-anti-HER3 mAb (GSK2849330) (164) (see also previous paragraph). In the ^89^Zr-anti-EGFR study patients received 500 mg/m^2^ (range = 870–1,040 mg) of unlabeled mAb followed by 37 MBq ^89^Zr-anti-EGFR (cetuximab) with 10 mg mass dose. Imaging was performed at 1–2 h, D_1_, D_2_, D_3,_ and D_6_ p.i. Analysis was based on 4 patients with a total of 7 tumors. For ^89^Zr-anti-EGFR, the residuals between SUV at D_6_ and Patlak were relatively small and improved when TPR or TBR were used instead of SUV. For the ^89^Zr-anti-HER3 data using different mass doses, the residuals were larger. Here, only TPR and TBR were reliable, whereas SUV was not. The results suggest that for these two mAbs, TPR and TBR of late imaging time points (D_5_ or D_6_) could provide valid quantification of irreversible ^89^Zr-immunoPET uptake. SUV was particularly unreliable for different mAb doses, as it does not account for patient-specific plasma clearance.

It should be noted that the articles reviewed in this subsection originate from the same research group.

#### 3.2.2 Tracer-kinetic modeling of a ^124^I-labeled mAb

Daghighian et al. (165) published (in 1993!) about kinetic modeling of ^124^I-3F8 in glioma with the aim of improving dosimetry for RIT. This proof of concept study consisted of one glioma patient injected with 96.2 MBq of tracer. Scans were performed immediately p.i. with frames of 8 × 5 and 2 × 25 min as well as at 18 h, 18.5 h, 66 h p.i., on D_10_ and on D_11_ p.i., with careful repositioning of the patient using a laser marker system. Plasma samples were obtained as well over the range of 11 days. The 2TCM was applied to estimate $K_{1}$, $k_{2}$, $k_{3}$, $k_{4}$, and the binding potential $\mathrm{BP}=k_{3}/k_{4}$. Tumor radioactivity was highest at 18 h p.i. Kinetic modeling allowed to calculate the amount of tumor-bound radiolabeled antibody, which permits to perform both macro- and microdosimetry using non-invasive imaging and may be applied for RIT.

#### 3.2.3 Non-linear compartmental modeling of ^124^I-A33

Zanzonico et al. (116) applied non-linear compartmental modeling to immunoPET data of ^124^I-labeled A33. Due to the saturability of the antibody-antigen interaction, a non-linear analysis was performed. Serial whole-body PET scans ranging from 3 h to D_9_ p.i. and blood samples were acquired. The starting values of the association rate constant, the total A33 concentration in normal bowel and the total A33 concentration in tumor were measured independently *in vitro*. TACs for each patient were fit to a non-linear compartment model resulting in excellent agreement between fitted and measured parameters of tumor uptake, off-target uptake in bowel mucosa, blood clearance, tumor antigen levels, and percent antigen occupancy.

The authors concluded that this approach should be generally applicable to antibody-antigen systems in human tumors for which masses of both antigen-expressing tumors and normal tissues can be estimated. They state that in this manner, a patient-specific optimum mAb dose may be derived. The authors also discussed the relevance of their methodology for RIT with the rationale that the concentration of A33 in targeted tumors, the total mass of a tumor and tumor-to-normal tissue ratios will vary widely between patients. Therefore, there is no single optimum antibody dose, and the authors suggested using imaging-based measurements to find the patient specific optimum dose for RIT, which yields the maximum tumor-to normal tissue AUC and thus the maximum tumor-to-normal absorbed-dose ratios.

#### 3.2.4 Tracer-kinetic modeling of ^89^Zr-minibody

Omidvari et al. (17) reported dynamic LAFOV PET imaging of the CD8-targeted minibody ^89^Zr-Df-IAB22M2C using tracer-kinetic modeling in healthy subjects and in COVID-19 convalescent patients. Imaging was performed dynamically over the first 90 min p.i. as well as at 6 h and 48 h p.i. for 60 min in the 194-cm-long uEXPLORER scanner, and with a relatively low injected activity dose of on average 18.8 MBq.

Conventional approaches based on one-tissue and two-tissue compartment models successfully fitted TACs in lungs, spleen, bone marrow, tonsils, and lymph nodes. In those organs, the slope of the Patlak plot changed from 90 min to 48 h. TBR (here: tissue-to-blood-ratio) curves and $K_{i}$ of the bone marrow of COVID-19 patients were elevated during the first 7 h of the study. The authors describe that these changes were not evident in the SUV images since they do not account for the time-varying tracer concentration in blood and tissue. However, they found that TBR from the 6 h time point was highly correlated with $K_{i}$ obtained from the full 48 h TACs, suggesting that at the right time point TBR might be used as a surrogate for $K_{i}$.

The authors also described the importance of cell trafficking for imaging of targets such as CD8+ T-cells. In the absence of cell trafficking, the main mechanism of uptake can be simplified by the 2TCM with $k_{4}=0$, where the ^89^Zr-labeled minibody binds to the receptor and is irreversibly internalized within the cell. However, with non-negligible cell trafficking and imaging over longer time periods, all rate constants need to include components from trafficking and a non-zero *k*_4_. The authors recommend future studies to investigate this aspect and incorporate it in the modeling approach.

Finally, the authors also discussed the effects of blood flow and blood vessel permeability. High blood flow regions with increased permeability allow for fast entrance of the tracer into the tissue, followed by a slower process of binding within the second tissue compartment. Whereas, in case of lower permeability and blood flow, such as in the lungs, a large fraction of the initial signal would be related to the blood volume fraction.

#### 3.2.5 Tracer-kinetic modeling of ^68^Ga-affibody

Alhuseinalkhudhur et al. (166) performed dynamic ^68^Ga-ABY-025 PET imaging of the upper abdomen with a 0–45 min p.i. scan in 16 metastatic breast cancer patients. Parametric images of $K_{1}$ and *K_i_* were created by employing an irreversible 2TCM and Patlak analysis together with an IDIF extracted from the descending aorta. A VOI-based analysis was performed to validate the parametric images. SUVs derived from the 2 h and 4 h p.i. static acquisitions were determined as well. Parametric imaging improved characterization of HER2 expression in smaller liver metastases. SUVs of metastases at 2 h and 4 h p.i. were highly correlated with $K_{i}$ values (*R*^2^ = 0.87 for 2TCM and 0.95 for Patlak).

### 3.3 Quantitative immunoPET in preclinical studies

Kim et al. (167) applied kinetic modeling in their ^89^Zr-CD4R1-F(ab')_2_ and ^89^Zr-ibalizumab study to image the CD4 pool (in view of HIV/SIV infection) in nonhuman primates. For the Fab fragment, a 4 h dynamic scan and a static scan at 40 h p.i. were performed. The authors applied full quantitative analysis including arterial sampling, metabolite evaluation, and model fitting to estimate the binding potential of CD4 receptors in the lymph nodes, spleen, and gut using a 2TCM and an IDIF. The authors found splenic SUV in an infected monkey to be higher than in uninfected controls but stress the semi-quantitative nature of SUV measurements that do not consider differential blood flow and clearance of radioligand between animals. When tissue uptake was adjusted for blood SUV, the spleen-to-blood uptake ratio was higher in uninfected than in SIV-infected animals. The authors also pointed out that for small tissues such as lymph nodes, SUV_mean_ suffered from partial volume effect by up to 40%. As no correction was applied, the binding potential of lymph nodes was assumed to be underestimated. The authors could estimate receptor densities, which, when translated from macaque to human levels, indicated that the relative size of the gut CD4 pool was lower than that of the splenic CD4 pool.

Aweda et al. (168) reported on the *in vivo* biodistribution and pharmacokinetics of sotrovimab, a SARS-CoV-2 monoclonal antibody, in healthy cynomolgus monkeys. In addition to TBR, they also applied a PBPK model to evaluate tissue biodistribution kinetics. They compared two similar mAbs, where one had a modified Fc region to extend its serum half-life. Results indeed showed an extended half-life of the mAb with a modified Fc region. PBPK modeling provided satisfactory fitting for most regions.

In a preclinical study Cheal et al. (169) compared ^89^Zr- with ^124^I-labeled cG250. Experiments with both tracers using a human clear-cell renal cell carcinoma cell line were performed to characterize binding affinity and internalization kinetics and serial PET imaging was performed in tumor-bearing mice. Equilibrium rates of antibody internalization and turnover in the tumors were derived from the PET images using nonlinear compartmental modeling. Both tracers showed identical tumor cell binding and internalization, but different retention *in vivo* with longer retention and superior PET images for ^89^Zr-cG250. The mean tumor concentration in mice was fitted using a nonlinear compartmental model and an IDIF obtained from a VOI on the heart region. Starting values for the association rate constant and the antigen concentration in tumor were measured *in vitro*.

Chevaleyre et al. (170) performed kinetic modeling of brain data in mice, applying focused ultrasound before performing an anti-PD-L1 immunoPET scan with ^89^Zr-DFO-C4 or its FcRn low-affinity mutant ^89^Zr-DFO-C4^Fc−MUT^. The latter tracer showed a significantly decreased efflux rate constant from healthy brain tissue to plasma compared with the non-mutated IgG. The authors concluded that this improves the kinetic properties since target engagement can be determined as early as 12 h p.i.

Laffon and Marthan (171) reported a three-time-point method for assessing kinetic parameters of a ^64^Cu-labeled mAb targeting VEGFR-2 positive lung tumors using data of tumor-bearing mice imaged at 3 h, D_1_ and D_2_ p.i. They fitted three parameters, namely $K_{i}$, a release rate constant $k_{r}$, and *F*, representing the fraction of free tracer in blood and interstitial volume to ACs determined with and without administration of a blocking dose. In another study, Laffon et al. (172) estimated kinetic parameters of ^64^Cu- and ^177^Lu-cetuximab using published mice data with regard to dosimetry prediction.

Another approach for determining specific uptake in tumors can be a dual-tracer approach. Cheng et al. (173) performed dual-tracer immunoPET imaging in mice with an ^11^C labeled EGFR-binding affibody molecule and its size-matched non-binding control. Both molecules showed similar biodistribution except for higher concentration of the non-binding control in liver and blood. The targeting tracer successfully visualized human squamous cell carcinoma with moderate to high EGFR expression levels. However, also non-specific uptake in tumors was high and sometimes equally large. There was no correlation between total EGFR and specific tracer uptake, which would indicate a discordance between available membranous and total EGFR expression levels.

In the preclinical study by Fung et al. (174) on the earlier mentioned tracer ^124^I-J591 the authors applied PBPK modeling and found a nonlinear model to be superior to the conventional linear model. They also compared with a ^89^Zr-labeled version of the tracer and found that the equilibrium constant was twice as high for ^124^I, but there was about a tenfold greater tumor efflux rate for ^124^I compared with ^89^Zr, while surface binding and internalization rates were similar.

Wilks et al. (175) published about improved modeling for kinetics of slowly diffusing radiotracers for tumor imaging using partial differential equations with Bayesian priors to model a radially symmetric reaction-diffusion equation describing immunoPET uptake. The model was applied to ^124^I-labeled A11 anti-prostate stem cell antigen minibody imaging in mice. The results showed estimates of the dissociation constant and receptor density close to *in vitro* measurements and also large differences with regular compartmental modeling that ignores tracer diffusion limitations.

### 3.4 Simulation studies

Simulation studies can inform clinical immunoPET protocols and may improve understanding of immunoPET data. Two types of modeling studies, PBPK modeling (134) and the conventional PET modeling, have distinct roles (e.g., 2TCM, Patlak).

Wijngaarden et al. (176) performed a simulation study regarding the optimal imaging time points when applying Patlak to ^89^Zr-immunoPET data. Tissue TACs were simulated based on three different ^89^Zr-mAb input functions and published reference values for reversible (*V_T_*) and irreversible (*K_i_*) uptake. The input functions were extracted from published studies with multiple blood samples on the day of injection and further blood samples with every PET scan. Accuracy and precision of Patlak linearization were evaluated by comparing simulated *K_i_* and *V_T_* with reference values for different imaging time points and noise levels.

For high *K_i_*, accuracy and precision of Patlak results decreased with smaller AUC of the input function (i.e., faster clearance) for the three different mAbs. The authors reasoned that for immunoPET tracers with faster kinetics, the data points will be closer together and the effect of noise would be stronger. Furthermore, they explained that limited sampling of the AIF can lead to an overestimation of the AUC_p_ and therefore to a negative bias in *K_i_*. They concluded that a blood sample at 24 h p.i. improves accuracy due to a better assessment of the shape of the AIF, but this depends on the kinetics of the tracer as steady-state equilibrium needs to be achieved at the first Patlak time point. In general, sampling at the most curved part of the IF was found to be critical. Further, the authors concluded that the interval of the two additional sampling points is not so critical. Similar principles may apply to other mAb based constructs. For example, in the ^89^Zr-minibody study by Omidvari et al. (17) a second scan on D_0_ was performed from 6 to 7 h, where kinetic changes in the blood pool are still high.

Huisman et al. (71) simulated ^89^Zr-trastuzumab immunoPET TACs using a PBPK model together with an input function and several trastuzumab parameter values reported in the literature. The relationship between PET uptake and concentration of HER2 receptors in a tumor was mathematically modelled. The uptake initially increased with the target concentrations, until it reached a constant value, which is determined by the total administered mass dose of trastuzumab. The results indicated that for 50 mg ^89^Zr-Trastuzumab, SUV can discriminate between IHC score 0 vs. 1, 2, and 3 but not among IHC 1, 2, or 3. The authors therefore conclude that although ^89^Zr-trastuzumab immunoPET can be used to assess target expression, there is a risk of false-positive results depending on the cut-off used to define target positivity and the dose administered.

Huisman's model includes the extravasation rate constant, which determines the extravasation rate for a given concentration gradient and combines the vasculature and the degree of vascularization of the tissue. The authors discussed that the extravasation rate constant is the key factor that determines the maximum tumor uptake at a late time point and varies between and within patients. Furthermore, they discussed that higher plasma clearance leads to lower SUV and therefore suggested that different plasma PK curves at different mass doses can be used to investigate this effect. The Huisman model does not include any blood flow parameter. Thurber and Wittrup (65) described that their PBPK model results indicated that in the case of intact mAbs, while vascularization is very important, the velocity of blood flow did not have a major impact due to the extremely slow rate of extravasation and lack of depletion along the length of the vessel. This may be different for smaller mAb based constructs.

Shah et al. (134) and Liu et al. (177) developed a platform PBPK model and validated the model using human ^89^Zr-immunoPET data. Liu et al. used immunoPET data from eight studies, but only two were used to validate the tumor PK model due to limited data in the literature because most studies did not report essential tumor-related parameters. To indirectly validate the tumor PK model, the authors used data from (158) with blood PK for four doses (100, 200, 450, and 675 mg), fitting them together with the PBPK model. Further validation was conducted using tumor PK data from a study in which patients were dosed with ^89^Zr labeled bevacizumab to predict the antibody distribution of tumors without target expression since bevacizumab does not target tumor cell surface antigens but rather VEGF in the tumor environment. The authors concluded that more clinical ^89^Zr immunoPET data in various antigen expression tumor types are needed to validate the tumor model. Once established, these models will significantly aid in quantitatively assessing antigen expression with ^89^Zr immuno-PET, potentially guiding personalized treatment.

## 4 Discussion

This article reviewed the methodology of immunoPET studies and the role of imaging protocols and quantification methods in determining how well immunoPET can assess target engagement and thus how well it can aid patient selection for a specific therapy and guide drug development. Many early stage immunoPET studies included multi-time point imaging in a small patient population. The most common goals of these studies were to demonstrate that they can provide safely and non-invasively an overview of both biodistribution and tumor targeting of the investigated tracer. While in the first part of these studies, multi-time point imaging was applied to find the best imaging time point for visualization and sometimes for dosimetry purposes, in the second part or follow up studies usually only one static scan was performed. In addition to oncological immunoPET, research is also being conducted into infectious and inflammatory diseases.

Many studies involve co-injecting a non-labeled, also referred to as “cold”, amount of the molecule to saturate non-specific uptake and improve visualization of lesions. As discussed by several articles, this may (partially) occupy receptors in the target regions (e.g., tumors) as well, which will even be more pronounced when patients undergo antibody therapy at the time of imaging the same target. With escalating cold dose, a decrease in target signal can be demonstrated in some cases suggesting target saturation. It needs to be considered that in conventional PET kinetic modeling, tracer amounts are assumed with linear kinetics. However, with larger amounts of cold molecules, there may be saturable binding, which would make it necessary to incorporate more complex non-linear models that will include modeling of the cold molecules. The rate of mAb binding to antigen will decrease as an increasing number of antigenic sites are already occupied.

The wide range of immunoPET tracers will need to apply different approaches in terms of PET quantification methodology. Full mAbs with long-lived radionuclides are likely to require multiple time-point imaging, whereas smaller molecules may use short-lived tracers with imaging on the same day. Small molecules are also expected to be more affected by perfusion effects as discussed by Omidvari et al. (17), whereas full mAbs may be less dependent on perfusion. In addition, T-cell tracers may be affected by T-cell trafficking. For relatively fast tracers, dynamic imaging can help in optimizing the best time window for scanning. Similarly for full mAb immunoPET multiple time-points over days may provide additional information about kinetics and image quality to determine the optimal imaging time-point.

Regarding semi-quantitative analysis, most studies consider manual delineation of organs and lesions combined with reporting SUV_max_ normalized to bodyweight, body surface area, or LBM. In general, the use of SUV is based on the idea of the distribution volume, where SUV_LBM_ is a recommended metric for FDG uptake (178), however the use of SUV for any immunoPET tracer is questionable in itself as normalization to body weight is not always a good measure of initial tracer distribution volume. A subset of studies reported TBR values normalizing SUV_max_ of the tumor to SUV_mean_ of a blood pool region to reduce the bias induced by different tracer availability. A few studies report SUV_peak_ when quantifying lesions instead of SUV_max_. From a PET methodology perspective, data-driven approaches improve the reliability of quantification measures compared with manual delineations (53). In addition, due to noise, SUV_peak_ is more appropriate for ^89^Zr-immunoPET imaging (51, 179, 180).

Demonstrating correlations between immunoPET and invasive biopsies is challenging, and only small sample sizes have been published, showing several limitations. Biopsies cannot access all lesions and can only give information about a small part of potentially heterogeneous disease. When correlating immunoPET metrics with biopsy, it is difficult to show whether uptake in target negative lesions is due to effects of heterogeneity or due to nonspecific uptake. In addition, even if there is high target expression, mAb distribution to parts of the target region, distant from functional vessels, is limited. In many cases, the assessment of target expression alone may not be sufficient to predict therapy response (181). This may be an argument in further investigating image-based markers as a predictor for treatment response that can relate to heterogeneity in lesions and longitudinal changes of receptor expression.

### 4.1 Static vs. dynamic immunoPET

The general controversy of using static or dynamic imaging (46) also comes into play with immunoPET. This is even more challenging due to slow kinetics of the molecules involved, which means that often multiple time-point scanning is necessary, leading to higher demands on scanner-time and additional efforts in image analysis. Static immunoPET offers a simpler approach, primarily focusing on the visualization and semi-quantification of lesions. However, this approach does not capture patient and lesion-dependent kinetics that may give additional information in the drug-development stage or in aiding decision-making or predicting treatment response. Dynamic or multi-time point immunoPET may provide additional information about the biology of the regions investigated. Especially with the small number of patients in phase 1 studies this additional information may be useful to differentiate specific from non-specific uptake and may increase the chance of finding correlations with biopsies or treatment response, and determining optimal imaging and treatment doses.

On the other hand, dynamic immunoPET presents challenges related to its practical implementation and kinetic analysis. It is challenging to select sufficient time points to properly characterize (and model) its kinetics, and in practice usually limited kinetic data are available. The Patlak plot only requires a few data points for the target tissue, but sufficient samples of the input function by using either an IDIF or blood samples. The articles reviewed indicated that the Patlak approach is feasible for VOIs and that it can provide additional information over semi-quantitative SUV. Although, in theory, two imaging time points would be sufficient for this linear approach, three or more may be required for validation, where the first point should be selected after reaching steady-state equilibrium between plasma and the non-displaceable compartment. If the initial distribution and/or an IDIF needs to be measured, a scan at time of injection might be used as well, leading to four scans, which subsequently can be reduced if e.g., initial patient data show that two late time-point images are sufficient to calculate the desired metrics. As discussed in the study by Wijngaarden et al. (175), for ^89^Zr-labeled mAbs, it is important to perform a scan or blood sample at D_1_ in order to capture the shape of the AIF. The two additional sampling points were found to be less critical. In case of of smaller mAb based constructs, like minibodies, a second scan at the day of injection, e.g., 6 h p.i., seems to be beneficial for capturing its kinetics [see also Figure 3 and Omidvari et al. (17)]. Finally, to capture kinetics of mAb-based constructs smaller than minibodies, long dynamic acquisitions immediately after injection would be useful.

Given the research gap identified in the present article, it is clear that that simplified metrics, such as SUV, need to be validated carefully against a multi-time point or dynamic approach (46), and therefore improved immunoPET quantification methodology is necessary. If a semi-quantitative metric like TBR turns out to be sufficient when compared to e.g., Patlak *K_i_*, in clinical practice a single time-point can be used (39).

### 4.2 The role of long axial field of view PET/CT

At present, there are only a few articles available that performed immunoPET on the recently introduced LAFOV scanners. Due to their increased FOV and sensitivity, these scanners lead to substantially higher image quality and can facilitate the use of IDIF and kinetic modeling techniques.

Mohr et al. (180) compared semi-quantitative immunoPET on a short vs. a LAFOV PET/CT system and demonstrated an eight-fold reduction in scan duration compared to a state of the art short axial field of view PET/CT. However, the authors recommend reducing only by a factor of 2–3 in order to maintain high image quality. Acquiring high quality image data is particularly important for dynamic imaging as performed in the previously described study of Omidvari et al. (17). Using LAFOV PET, they could investigate high-quality dynamic imaging of a CD8+-minibody at radiation doses lower than 10 mSv, allowing for longitudinal imaging of non-oncological patients or even healthy subjects.

In addition to shortening scan duration or reducing radiation dose, the higher sensitivity can also be used to perform imaging at delayed time points (91). This is particularly promising because in case of irreversible tracers like ^89^Zr-labeled mAbs, the later the imaging time point, the more accumulation of irreversible signal and decrease of reversible (non-specific) signal can be expected. The reduced scan durations with LAFOV PET make multiple time-point scanning and delayed imaging time points more realistic even in a busy clinic.

### 4.3 Other aspects of multi-time point image analysis

When comparing uptake values from scans on different days, the quantitative parameters reported should be derived using the same VOI method. ImmunoPET often involves scanning on different days, which in turn requires delineating the same regions on images obtained in different positions. Segmentation of a VOI can either be performed on the CT images or on the PET images themselves. This is, however, associated with several challenges regarding the consistency and accuracy of the segmentations, as a different VOI may be derived at each scan time point. For segmentation performed on PET images, additional challenges include a PET signal that changes over time, potentially heterogeneous lesions and increased image noise in later acquisitions. Therefore, segmentation on CT scans may be preferred, but this in turn may be limited by the fact that voxels will be segmented based on anatomical characteristics (tissue density) rather than on molecular characteristics that are relevant for PET. In addition, potential misalignments may exist between corresponding regions on CT and PET scans. Ultimately, all segmentation techniques may suffer from inter- and/or intraobserver variability.

Another aspect is that multi-time point imaging requires multiple CT scans that increase the overall radiation dose. There are several approaches that avoid CT altogether and still provide an attenuation map for attenuation correction, e.g., using the LSO background of the scanner (182), or using a simultaneous reconstruction of activity and attenuation map from the PET data [MLAA (183, 184)]. Alternatively, it is possible to perform an ultra-low dose CT by using a tin filter (185) and/or adjusting CT settings (186). However, without at least one CT of sufficient quality, segmentation will be difficult. Another important factor is the effect of patient motion, leading to both, a mismatch between CT and PET scan affecting attenuation correction (187) and to blurred structures in the PET images (188). It should be noted that motion correction was not applied in any clinical immunoPET studies published so far.

If registration and motion correction could be solved with high performance, there would be potential for voxel-wise kinetic modeling leading to parametric immunoPET images as used in more conventional dynamic PET scans (189–191). This approach, however, will be more feasible for smaller mAb-based constructs in combination with shorter-lived radionuclides that can make use of a dynamic scan protocol or in mAb studies focusing on areas of the body that can be registered more easily (e.g., rigid motion of the head). In addition, parametric immunoPET images would be easier for $K_{i}$ than for microparameters due to noise. So far, only one study could be found that performed parametric imaging for an immunoPET application [^68^Ga-ABY-025 affibody (166)].

Beyond SUV_max_ and SUV_peak_, other image features obtained by radiomics (192) may be applied to immunoPET. For instance, a more homogeneous target expression may be related to better treatment response, while heterogeneous signals may correlate with poorer response, as demonstrated in a preclinical study by Rashidian et al. (193) using an anti-CD8 antibody fragment tracer. Therefore, radiomics may have a role in predicting therapy response and may benefit from decreased image noise of LAFOV PET/CT scanners (194).

### 4.4 Dual-tracer approaches

Dual-tracer approaches are emerging that allow for measuring simultaneously two different radiotracers (and corresponding biological processes) that conventionally are imaged sequentially (195) reducing the burden for the patient. This approach might also be useful to complement the information of an immunoPET scan by combining one of the multiple time point scans with a different tracer. Commonly performed ^18^F-FDG scans in patients that will receive an immunoPET scan could be combined and may help to predict treatment response (196). In addition, the information of ^15^O-H_2_O PET to measure perfusion may be used to understand and correct the confounding effect of perfusion on PET metrics like *K_i_*. A dual immunoPET approach to measure the status of two different immune-related targets might also be helpful and can be achieved by using two different radionuclides like ^124^I and ^89^Zr that can be discriminated via the additional gamma photon emitted by ^124^I (197). By applying one targeted and one untargeted tracer with similar transport kinetics, BP may also be estimated using a dual tracer approach instead of conventional kinetic modeling (198).

## 5 Conclusions

Most immunoPET studies have been analyzed semi-quantitatively. A subset of these studies have pointed out that SUV cannot capture patient-dependent plasma clearance and that it may be affected by disease-dependent characteristics and treatment-related factors. A handful of studies have demonstrated the potential use of Patlak in immunoPET. Several challenges exist in moving beyond semi-quantitative immunoPET since long dynamic or multiple time-point scans are needed together with more complex analyses. However, dynamic immunoPET imaging is currently the only available non-invasive technology that can provide *in vivo* insights into highly specific receptor binding or immune cell distribution throughout the body. This is a research gap that may, if resolved, have the potential to increase the power of immunoPET. New or continuing studies with larger patient cohorts, combined with recent advances in PET scanner technology, hold a promise of developing more reliable immunoPET imaging methodology.
