# Supplementary material for: Advances and challenges in immunoPET methodology
Source: Front Nucl Med. 2024 Feb 19;4:1360710. doi: 10.3389/fnume.2024.1360710 (PMC11440922; doi:10.3389/fnume.2024.1360710)
Supplement: Supplementary file 1 [file Table1.docx]

Supplementary Material

Advances and challenges in immunoPET methodology

Philipp Mohr^1*^, Joyce van Sluis^1^, Marjolijn N. Lub-de Hooge^1,2^, Adriaan A. Lammertsma^1^, Adrienne H. Brouwers^1^, Charalampos Tsoumpas^1^

^1^ University of Groningen, University Medical Center Groningen, Department of Nuclear Medicine and Molecular Imaging, Groningen, Netherlands

^2^ University of Groningen, University Medical Center Groningen, Department of Clinical Pharmacy and Pharmacology, Groningen, The Netherlands

**^*^Correspondence:** p.mohr@umcg.nl

**Literature search**

The search engines PubMED and Embase and Web Of Science Core Collection were consulted for relevant articles until January 6, 2024 without restricting the start date. For each database a search string was constructed including terms like immuno-PET and immunoPET. However, since not all studies call radiolabeled mAb-based PET imaging immunoPET, the search strings included combinations of different names for mAb constructs (e.g. mAb, F(ab), minibod*, nanobod* etc.) and PET or the corresponding MeSH terms for Monoclonal Antibodies and Positron-Emission-Tomography. All reference lists of relevant articles were cross-checked for additional literature.

**PubMed:**

(Immuno-pet[tiab] OR immunoPET[tiab] OR mAb[tiab] OR antibod*[tiab] OR IgG[tiab] OR "F(ab)"[tiab] OR Fab[tiab] OR minibod*[tiab] OR ScFv[tiab] OR fragment*[tiab] OR nanobod*[tiab] OR affibod*[tiab] OR diabod*[tiab] OR "Antibodies, Monoclonal"[Mesh] OR "Zr"[tiab]) AND (PET[tiab] OR positron*[tiab] OR "Positron-Emission Tomography"[Mesh]) AND (patient*[tiab] OR subject*[tiab] OR women[tiab] OR men[tiab] OR primate*[tiab] OR monkey*[tiab] OR individuals[tiab]) NOT (Review[Publication Type] OR Systematic Review[Publication Type])

Embase:

('Immuno-pet':ab,ti,kw OR immunoPET:ab,ti,kw OR mAb:ab,ti,kw OR antibod*:ab,ti,kw OR IgG:ab,ti,kw OR 'F(ab) ':ab,ti,kw OR Fab:ab,ti,kw OR minibod*:ab,ti,kw OR ScFv:ab,ti,kw OR fragment*:ab,ti,kw OR nanobod*:ab,ti,kw OR affibod*:ab,ti,kw OR diabod*:ab,ti,kw OR 'monoclonal antibody'/mj OR 'Zr':ab,ti,kw) AND (PET:ab,ti,kw OR positron*:ab,ti,kw OR 'positron emission tomography'/exp) AND (patient*:ab,ti,kw OR subject*:ab,ti,kw OR women:ab,ti,kw OR men:ab,ti,kw OR primate*:ab,ti,kw OR monkey*:ab,ti,kw OR individuals) NOT (‘conference abstract’/it OR ‘review’/it OR 'systematic review'/exp)

WOS Core Collection:

TS=(Immuno-pet OR immunoPET OR mAb OR antibod* OR IgG OR "F(ab)" OR Fab OR minibod* OR ScFv OR fragment* OR nanobod* OR affibod* OR diabod*) AND TS=(PET OR positron*) AND TS=(patient* OR subject* OR women OR men OR primate* OR monkey*) NOT DT==("REVIEW" OR "MEETING ABSTRACT")

Additional PubMed search string with modelling terms and without patient term:

(Immuno-pet[tiab] OR immunoPET[tiab] OR mAb[tiab] OR antibod*[tiab] OR IgG[tiab] OR "F(ab)"[tiab] OR Fab[tiab] OR minibod*[tiab] OR ScFv[tiab] OR fragment*[tiab] OR nanobod*[tiab] OR affibod*[tiab] OR diabod*[tiab] OR "Antibodies, Monoclonal"[Mesh] OR "Zr"[tiab]) AND (PET[tiab] OR positron*[tiab] OR "Positron-Emission Tomography"[Mesh]) AND (kinetic[tiab] OR Patlak[tiab] OR compartment*[tiab]) NOT (Review[Publication Type] OR Systematic Review[Publication Type])

**Supplementary Table 1.** Overview of semi-quantitative clinal immunoPET studies using intact mAbs.

| Publication (year) (reference number in manuscript) | Study cohort | Tracer  (target) | Average Activity, Total protein doses | Imaging time points | (Semi-) quantification metrics | Correlation PET uptake to target expression in biopsies | Correlation PET uptake to response |
| --- | --- | --- | --- | --- | --- | --- | --- |
| Dijkers et al. (2010) (72) | Breast cancer (n = 14) | ^89^Zr-trastuzumab (HER2) | 37 MBq,  50 mg treatment-naive/10 mg on treatment | D_1-3_, D_4-7_ | Mean relative uptake value (tissue/whole-body) | - | - |
| Ulaner et al. (2020) (73) | Multiple myeloma (n = 10) | ^89^Zr-DFO-daratumumab (CD38) | 74 MBq, 3/20/50 mg | D_1_, D_2-4_, D_7-8_ | SUV_max_ | - | - |
| Muylle et al. (2015) (74) | B-cell lymphoma (n = 5) | ^89^Zr-rituximab (CD20) | 111 MBq, tracer-only/250 mg/m^2^ preload | 1 h, D_3_, D_6_ | SUV_max_ (lesions) | - | - |
| Niemeijer et al. (2022) (75) | NSCLC (n = 12) | ^89^Zr-pembrolizumab (PD-1) | 37 MBq,  2 mg, 200 mg preload | 1 h, D_3_, D_5_, D_7_ (first 3 patients), D_3_, D_6_ (others) | SUV_mean_ (healthy tissues),  SUV_peak_ (lesions) | No significant correlation | No significant correlation |
| Smit et al. (2021) (76) | NSCLC (n = 13) | ^89^Zr-durvalumab (PD-L1) | 37 MBq, 2 mg, 750 mg preload | 1 h, D_3_, D_5_, D_7_ (first 3 patients), D_3_, D_7_ others) | SUV_mean_ (healthy tissues), SUV_peak_ (lesions) | No significant correlation | No significant correlation |
| Lohrmann et al. (2019) (77) | Pancreatic cancer (n = 12) | ^89^Zr-MVT-2163 (CA19-9) | 169 MBq,  3/20/50 mg | D_0_, D_1_, D_2-4_, D_5-7_ | SUV_mean_ (healthy tissues),  SUV_max_ (lesions) | - | - |
| Bensch et al. (2017) (78) | Different cancer types  (n = 20) | ^89^Zr-lumretuzumab (HER3) | 37 MBq, Part A: 11/51/101 mg.  Part B: 101 mg + 400/800/1600 mg | Part A: D_2_, D_4_, D_7_. Part B: D_4_, D_7_ | SUV_mean_ (healthy tissues);  SUV_max_, TBR (lesions) | No significant correlation | No significant correlation |
| Lindenberg et al. (2017) (79) | Colon cancer (n = 3) | ^89^Zr-panitumumab (EGFR) | 37 MBq | 2-6 h, D_1-3_, D_5-7_ | Activity amount (dosimetry) | - | - |
| Beckford-Vera et al. (2022) (80) | HIV (n = 10)  Control (n = 5) | ^89^Zr-VRC01 (CD4bs on HIV-1) | 37 MBq, 1 mg | 2-3 h/4-6 h, D_1_, D_3_, D_6_ | SUV_max_, SUV_mean_,  TBR | Correlation TBR & p24 levels in lymph nodes | - |
| Laforest et al. (2016) (82) | Breast cancer  (n = 12) | ^89^Zr-trastuzumab (HER2) | 62 MBq, 50 mg treatment-naive/10 mg on treatment | D_1-3_, D_2-5_, D_4-6_ | Visual assessment | - | - |
| Pandit-Taskar et al. (2014) (83) | Prostate cancer (n = 10) | ^89^Zr-huJ591 (PSMA) | 191 MBq, 25 mg | 2-4 h, D_1_, D_2-5_ | SUV_LBM-max_ (lesions) | 12 biopsy positive lesions, one not positive on PET | - |
| Pandit-Taskar et al. (2015) (84) | Prostate cancer (n = 50) | ^89^Zr-huJ59 (PSMA) | 185 MBq, 25 mg | First 10 patients: 2-4 h, D_1_, D_2-5_;  Others: D_6-8_ | Visual assessment, SUV_max_ (lesions) | Bone lesions: 17/21 positive on biopsy, 19/21 positive on PET  Soft-tissue lesions: 22/25 positive on biopsy, 14/22 positive on PET | - |
| O’Donoghue et al. (2018) (85) | Esophagogastric adenocarcinoma  (n = 10) | ^89^Zr-trastuzumab (HER2) | 184 MBq, 50 mg | 4 h, D_1_, D_2-4_, D_5-8_ | SUV_LBM,mean_ (healthy tissues), SUV_LBM,max_ (lesions) | - | - |
| Ulaner et al. (2018) (86) | Breast cancer (n = 6) | ^89^Zr-pertuzumab (HER2) | 74 MBq,  20/50 mg | D_1_, D_2-4_,  D_5-6_, D_7-8_ | SUV_LBM,mean_ (healthy tissues), SUV_LBM,max_ (lesions) | - | - |
| Shujing et al. (2021) (87) | Gastric cancer (n = 9), Colorectal cancer (n = 12) | ^89^Zr-CTB006 (DR5) | 52.8 MBq | First 6 patients:  2 h, D_1_, D_2_, D_3_, D_5;_ Others: 2 h, D_2_, D_3-4_, D_5_ | SUV_max,_ SUV_ratio_ (tumor: adrenal glands) | PET uptake in patients with RNAscope scores of 3-4 significantly higher than with scores of 0-2 | - |
| Jansen et al. (2017) (88) | Glioma  (n = 7) | ^89^Zr-bevacizumab (VEGF) | 0.9 MBq/kg, 0.1 mg/kg | 1 h, D_3_, D_6_ | SUV_mean_ (lesions), tumor-to-blood-ratio | - | - |
| O’Donoghue et al. (2019) (89) | Prostate cancer  (n = 7) | ^89^Zr-MSTP2109A (STEAP1) | 184 MBq, 10 mg | 1-4h, D_1_, D_2-3_, D_5-7_ | SUV_LBM,mean_ (healthy tissues)  SUV_LBM,max_ (blood pool, lesions) | - | - |
| Bensch et al. (2018) (93) | Breast cancer (n = 20) | ^89^Zr-trastuzumab (HER2) | 37 MBq, 50 mg | D_4_ | SUV_mean_ (healthy tissues),  SUV_max_ (lesions) | - | - |
| Bahce et al. (2014) (94) | NSCLC  (n = 7) | ^89^Zr-bevacizumab (VEGF) | 36.4 MBq, 5 mg | D_4_, D_7_ | SUV_peak_ (lesions) | - | No significant correlation for SUV_peak_ with response |
| Dehdashti et al. (2018) (95) | Breast cancer  (n = 50) | ^89^Zr-trastuzumab (HER2) | 76.6 MBq, 50 mg treatment-naive/10 mg on treatment | D_3-7_ | SUV_max_ (lesions) | SUV_max_ higher in HER2+ patients compared to HER2- patients when excluding hepatic lesions | - |
| Niemeijer et al. (2018) (96) | NSCLC (n = 13) | ^89^Zr-nivolumab (PD-1) | 37 MBq, 2 mg | First 3 patients:  1 h, D_3_, D_5_, D_7_  Others: D_7_ | SUV_peak_ (lesions) | Lesions with no PD-1 expression have lower SUV_peak_ than lesions with expression | SUV_peak_ of responding patients higher than for non-responders |
| Kok et al. (2021) (97) | Melanoma (n = 11) NSCLC (n = 7) | ^89^Zr-pembrolizumab (PD-1) | 37 MBq, 5/10 mg | D_2_, D_4_, D_7_ | SUV_mean_  (healthy tissues),  SUV_max_ (lesions) | PD-1 expression of IHC did not correlate with geometric mean SUV_max_ per patient | SUV_max_ correlated with tumor response & progression-free & overall survival |
| Ulaner et al. (2017) (98) | HER2-negative primary breast cancer  (n = 11) | ^89^Zr-trastuzumab (HER2) | 185 MBq, 50 mg | D_5-6_ | SUV_max_ (lesions) | 4 patients positive on PET, 1 biopsy-proven HER2+ metastases, 3 false positive | - |
| Even et al. (2017) (99) | Head and neck cancer (n = 17) | ^89^Zr-cetuximab (EGFR) | 54.5 MBq, 10 mg | D_3-4_, D_6-7_ | SUV_peak_, TBR, SUV_mean_ | SUV_mean_ & SUV_peak_ different between low & high EGFR, TBR_mean_ & TBR_peak_ not | - |
| Jauw et al. (2017) (100) | Diffuse large B cell lymphoma (n = 6) | ^89^Zr-rituximab (CD20) | 74 MBq, 10 mg, after first therapeutic dose of 700-1000 mg | D_0_, D_3_, D_6_ | SUV_peak_, SUV_mean_, TBR,  SUV_peak_ D6/D3 ratios | PET uptake & CD20 IHC concordant in 5 patients, in one patient PET positive, while biopsy negative | - |
| Bensch et al. (2018) (102) | Bladder cancer, NSCLC or triple-negative breast cancer (n = 22) | ^89^Zr-atezolizumab (PD-L1) | 37 MBq, 11 mg | 1 h, D_2_, D_4_, D_7_ | SUV_max_, tumor-to-background (lung & bone), TBR | Responses were better correlated with geometric SUV_max_ than with IHC- or RNA-sequencing-based predictive biomarkers | |
| Gaykema et al. (2014) (103) | HER2+ breast cancer  (n = 10)  ER+ breast cancer  (n = 6) | ^89^Zr-trastuzumab (HER2),  ^89^Zr-bevacizumab (VEGF) | trastuzumab: 37 MBq/50mg,  bevacizumab: 37 MBq/5 mg | D_2_, D_4_ for both tracers | SUV_mean_ (healthy tissues),  SUV_max_ (lesions) | - | ^89^Zr-trastuzumab SUV_max_ change related to size change on CT. |
| van Helden et al. (2020) (104) | RAS wild-type metastatic colorectal cancer  (n = 35) | ^89^Zr-cetuximab (EGFR) | 37 MBq, 10 mg after therapeutic pre-dose (500 mg/m^2^) | D_6_ | SUV_mean,_  SUV_peak_ | Tumor lesions with EGFR score > 10 higher SUV_peak_ than score < 10 | PET failed to predict treatment benefit |
| Ulaner et al. (2020) (105) | HER2-negative primary breast cancer  (n = 24) | ^89^Zr-pertuzumab (HER2) | 74 MBq, 50 mg | D_5-6_ | Visual analysis with five-point scale, SUV | 6 patients suspicious PET uptake, of which 3 biopsy-proven HER2+ metastases, 2 negative, 1 inconclusive | - |
| Merkx et al. (2021) (106) | Suspected clear cell renal cell carcinoma  (n = 10) | ^89^Zr-girentuximab | 37 Mbq, 5 mg/10 mg | 0.5 h, 4h, D_1_, D_3_, D_7_ | Visual analysis, Activity amount (dosimetry) | PET could differentiate between ccRCC and non-ccRCC lesions confirmed with biopsies | - |
| Lamberts et al. (2016) (107) | Pancreatic cancer  (n = 7)  ovarian cancer  (n = 4) | ^89^Zr-MMOT0530A (mesothelin) | 37 MBq, 1mg (2 patients), others: 10 mg | D_2_, D_4_, D_7_ | SUV_max_, TBR | Mesothelin expression on archival tumor tissue did not correlate with PET | - |
| Menke-van der Houven van Oordt et al. (2015) (108) | Colorectal cancer (n = 10) | ^89^Zr-cetuximab (EGFR) | 37 MBq, 10 mg, after first administration of 500 mg/m^2^ | 1-2 h, D_1_, D_2_, D_3_, D_6;_  3 Patients: D_6,_ D_10_ | SUV_peak_ (lesions), SUV_mean_ (lesions without visible uptake) | - | 6 patients were PET positive, of which 4 had clinical benefit, while progressive disease in 3 of 4 patients without uptake |
| Carrasquillo et al. (2019) (109) | Prostate cancer (n = 19) | ^89^Zr-DFO-MSTP2109A (STEAP1) | 185 MBq, 10 mg | D_4-7_ | SUV_max_, SUV_peak_, SUV_mean_ | 16 of 17 lesions biopsied were positive on PET, all histologically positive. No correlation of SUV_max_ & STEAP1 IHC | No correlation of PET with response |
| Van Es et al. (2017) (110) | Renal cell carcinoma  (n = 13) | ^89^Zr-bevacizumab (VEGF) | 37 MBq, 5 mg | D_4_ | SUV_mean_ (healthy tissues),  SUV_max_ (lesions) | - | Correlation between baseline tumor SUV_max_ & time on treatment |
| Verhoeff et al. (2022) (111) | Head and neck cancer  (n = 33) | ^89^Zr-Durvalumab (PD-L1) | 37 MBq, 2/10/50 mg | D_5_ | SUV_peak_, TBR | Uptake did not correlate to PD-L1 combined positive score of IHC | No correlation between uptake and response |
| Den Hollander (2015) (112) | Glioma  (n = 12) | ^89^Zr-Fresolimumab (TGF-β) | 37 MBq, 5 mg | D_2_, D_4_, or only D_4_ | SUV_mean_ (healthy tissues),  SUV_max_ (lesions) | - | No correlation between tumor uptake & PFS or overall survival |
| Rizvi et al. (2012) (150) | B-cell non-Hodgkin’s lymphoma  (n = 7) | ^89^Zr-ibritumomab tiuxetan (CD20) | 70 MBq | 1 h, D_3_, D_6_ | AC_mean_, %ID, SUV | - | - |
| Van Brummelen et al. (2018) (154) | CEA+ or CEA- solid tumors  (n = 24) | ^89^Zr-CEA-IL2v (immunocytokine targeting CEA) | 50 MBq, 2 mg | 2 h (in 3 patients), D_1_, D_4_, D_8_. Repeat scan in 4 patients with D_1_ and D_4_ | %ID/mL_mean_ (healthy tissues) %ID/mL_peak_ (lesions) | - | No relation between uptake & response |
| Mileva et al. (2024) (156) | Breast cancer  (n = 90) | ^89^Zr-trastuzumab (HER2) | 37 MBq, 50 mg | D_4_ | Visual classification in HER2-positive or HER2-negative | - | Combining HER2-PET/CT alone or in combination with FDG-PET/CT predicted response |
| Bruijnen et al. (2016) (12) | Rheumatoid arthritis  (n = 20) | ^89^Zr-rituximab (CD20) | 18 MBq, 10 mg, after therapeutic 1000 mg rituximab | D_3_, additionally D_6_ in 6 patients | Visual analysis, SUV_mean_ (wrist/hands), Target-to-background ratio (metacarpal bone) | CD22+ B-cell count in lymph node tissue at 4 weeks of treatment correlated inversely with PET uptake in hand joint and positively with lymph node uptake at baseline | Responders had higher uptake in PET-positive hand joints than non-responders |
| Hagens et al. (2018) (13) | Multiple sclerosis (n = 3) | ^89^Zr-rituximab (CD20) | 37 MBq, 10 mg after therapeutic dose of 1000 mg | D_1_, D_3_, D_6_ | Activity concentration | - | - |
| Adams et al. (2019) (14) | Pneumonitis  (n = 10) | ^89^Zr-rituximab (CD20) | 18 MBq, 10 mg after therapeutic dose of 1000 mg | D_3_, D_6_ | SUV_mean_, SUV_max_, TBR_max_, TBR_mean_ | - | - |
| Laban et al. (2019) (15) | Orbital inflammatory disease  (n = 12) | ^89^Zr-rituximab (CD20) | 74 MBq, 10 mg, | D_3_ | SUV_LBM,max_ | - | - |
| Börjesson et al. (2006) (29) | Head and neck cancer  (n = 20) | ^89^Zr-cmAb U36(CD44) | 75 MBq, 10 mg | 1 h, D_1_, D_3_, D_6_ | Visual analysis | Diagnostic sensitivity & accuracy comparable to FDG PET | - |
| De Ruijter et al. (2021) (90) | Different cancer types (n = 8) | ^89^Zr-CX-072 (PD-L1) Probody | 37 MBq, 1/5/10 mg | D_2_, D_4_, D_7_ | SUV_mean_ (healthy tissues), SUV_max_(lesions) | - | - |
| Guo et al. (2020) (113) | Gastric cancer  (n = 6) | ^124^I-trastuzumab (HER2) | 74 MBq, 5/10 mg | 1 patient: 1 h, D_1_, D_2_, D_3_, D_4_  Others: 1 h, D_1_, D_2_ | SUV_mean_,  SUV_max_ | - | - |
| Carrasquillo et al. (2011) (114) | Colorectal cancer  (n = 25) | ^124^I-huA33 (A33) | 343 MBq, 10 mg | 3 h, D_2_, D_5-9_ | SUV_mean_ (healthy tissues),  SUV_max_ (lesions) | - | - |
| O’Donoghue et al. (2011) (115) | Colorectal cancer  (n = 15) | ^124^I-huA33 (A33) | 200 MBq, 10 mg | D_5-9_ | Activity concentration | Amount of bound antibody proportional with antigen concentration determined in surgically removed tissue | - |
| Carrasquillo et al. (2018) (117) | Hepatocellular cancer  (n = 13) | ^124^I-codrituzumab (GPC3) | 185 MBq, 10 mg.  Repeat imaging after sorafenib/immunotherapy with 2.5 or 5 mg/kg codrituzumab | 1-4 h, D_1_, D_2/3_, D_4/5/6_ | SUV_mean,_ SUV_max_ (healthy tissues); SUV_max_ (lesions) | No significant correlation between SUV_max_ & IHC | No correlation between SUV_max_ & change in tumor volume |
| Pandit-Taskar et al. (2019) (151) | Metastatic leptomeningeal disease (n = 42) | ^124^I-omburtamab (B7-H3) | 74 MBq, 1 mg | 4 h, D_1_, D_2_ | Activity amount (dosimetry) | - | - |
| Grkovski et al. (2022) (152) | Desmoplastic small round cell tumors  (n = 31) | ^124^I-omburtamab (B7-H3) | 74 MBq, 1 mg | 4 h, D_1_, D_2_, D_3_, D_4_ | SUV_mean_ (healthy tissues) | - | - |
| Carrasquillo et al. (2019) (122) | Breast cancer (n = 11) | ^64^Cu-trastuzumab (HER2) | 296-370 MBq, 5 mg. | 1 h, D_1_ | SUV_LBM,mean_ SUV_LBM,max_ | - | - |
| Mortimer et al. (2018) (118) | HER2+ breast cancer (n = 8) | ^64^Cu-trastuzumab (HER2) | 364-512 MBq, 5 mg (for 2 patients)/50 mg (for others) | D_1_, D_2_ | SUV_mean_ (healthy tissues)  SUV_max_ (lesions) | - | - |
| Mortimer et al. (2018) (119) | HER2+ (n = 11), and HER2-  (n = 7)  breast cancer | ^64^Cu-trastuzumab (HER2) | 364-551 MBq (mean 464), 5 mg (for 2 patients) 50 mg (for others) | D_1_, D_2_ | SUV_mean_ (ventricle),  SUV_max_ (lesions) | SUV_max_ on lesion & patient level higher for HER2+ than HER2- | - |
| Kurihara et al. (2015) (121) | Patients with brain metastases from HER2+ breast cancer | ^64^Cu-trastuzumab (HER2) | 130 MBq, 74.4 µg | D_1_, D_2_ | SUV_max_ (lesions), Tumor-to-background ratio (normal brain) | - | - |
| Lockhart et al. (2016) (123) | Different cancer types (n = 11) | ^64^Cu-patritumab (HER3) | 296-555 MBq, ≤ 0.2 mg. 3 patients with uptake repeat scan week later with 9.0mg/kg therapy dose before tracer | 3 h, D_1_, D_2_ (dosimetry subjects),  D1 (others) | SUV_max,_  SUV_mean,_  TBR, Tumor-to-background (liver) ratio | No significant correlation between IHC & tumor uptake (SUV_max_ or tumor-to-blood ratio) | - |
| Krishnan et al. (2020) (124) | Multiple myeloma (n = 12) | ^64^Cu-daratumumab (CD38) | 555 MBq, 5/15/50/100 mg | D_0_, D_1_, D_2_ | SUV_mean_, SUV_max_ | 2 FDG positive lesions were negative with daratumumab & biopsy. FDG negative lesions but positive with daratumumab & biopsy | - |
| Wong et al. (2023) (125) | Patients with CEA-expressing primary or metastatic cancer (n = 20) | ^64^Cu-hT84.66-M5A (carcinoembryonic antigen CEA) | 555 MBq, 3 mg. | D_1_, D_2_ | SUV_mean_ (healthy tissues), SUV_max_ , TBR (lesions) | High concordance between ^64^Cu-DOTA-hT84.66-M5A PET & biopsies | - |
| McMahon (2021) (126) | HIV viremic (n=6)  HIV aviremic (n=2)  Control (n=4) | ^64^Cu-3BNC117 (CD4bs on HIV-1) | 120-130 MBq. 5 mg (tracer only subjects). Other subjects additional 3 mg/kg | PET/MR: 1 h, D_1_, D_2_ | SUV | - | - |

**Supplementary Table 2.** Overview of semi-quantitative clinal immunoPET studies using smaller mAb based constructs.

| Publication (year)  (reference number in main manuscript) | Study cohort | Tracer (type, target) | Average Radioactivity; Total protein doses | Imaging time points | (Semi-) quantification metrics | Correlation to Biopsies | Correlation to Response |
| --- | --- | --- | --- | --- | --- | --- | --- |
| Pandit-Taskar et al. (2016) (127) | Prostate cancer (n = 18) | ^89^Zr-IAB2m (minibody, PSMA) | 188.7,  10/20/50 mg | D_0_, D_1_, D_2/3_, D_4-5_ | SUV_LBM,mean_ (healthy tissues), SUV_LBM,max_ (lesions) | Lesions: 4 of 4 true-positive in bone, 1 true-negative in bone; 6 of 7 true-positive in soft-tissue | - |
| Pandit-Taskar et al. (2020) (128)  Farwell et al. (2022) (129) | Different cancer types (n = 15) | ^89^Zr-Df-IAB22M2C (minibody, CD8+) | 111 MBq, 0.2/0.5/1.0/1.5/5/10 mg | 2-4 h, D_1_, D_2_, D_4/5/6_ | SUV_mean_ (healthy tissues); SUV_max_, SUV_peak_ (lesions) | - | - |
| Schwenck et al. (2023) (130) | Different cancer types (n = 8) | ^89^Zr-Df-IAB22M2C (minibody, CD8+) | 74 MBq, 1.1-1.8 mg | PET/MRI D_1_ | SUV_mean_ | - | Hypothesis that alterations in lymphoid organs uptake associated with response |
| Moek et al. (2019) (131) | Gastrointestinal adenocarcinoma (n = 9) | ^89^Zr-AMG 211 (55 kDa bispecific T-cell engager, CEA) | 37 MBq, 0.2/2/5 mg | 6 h, D_1_, D_2_ (first patient), others: 3 h, 6 h, D_1_ | SUV_mean_ (healthy tissues),  SUV_max_ (lesions) | - | - |
| Kist de Ruijter et al. (2022) (132) | Different cancer types (n = 38) | ^89^ZED88082A (one-armed antibody, CD8+) | 37 MBq,  4/10 mg | 1 h, D_2,_ D_4_, D_7_ | SUV_mean_ (healthy tissues),  SUV_max_ (lesions) | Uptake correlated with CD8 IHC & autoradiography | Higher SUV_max_ correlated with longer overall survival |
| Thorneloe et al. (2019) (133) | Healthy subjects (n = 8) | ^89^Zr-GSK3128349 (AlbudAb, albumin-binding domain antibody) | 15 MBq/mg,  1 mg | 4 scans up to D_7_ | SUV_mean_ (healthy tissues)  SUV_peak_ (small healthy tissues) | - | - |
| Heuveling et al. (2013) (135) | Head and neck cancer (n = 4) | ^124^I-F16SIP (mini antibody  F16SIP 80kDa, extradomain B of fibronectin) | 74 MBq, 2 mg | 30 min and D_1_ | %ID/kg | - | - |
| Laforest et al. (2017) (136) | Different cancer types (n = 11) | ^124^I-PGN650 (F(ab’)_2_ fragment, pho-sphatidylserine) | 140 MBq, 1 mg | 1, 3 h and D_1/2_ | SUV_max_,  TBR | - | - |
| Scott et al. (2020) (137) | Prostate or ovarian  cancer (n = 5) | ^124^I- PEG-AVP045 (diabody, AG-72 glycoprotein) | 111-185 MBq,  1 or 10 mg/m2 | D_0_, D_1_, D_2/3_, D_4/5_, D_6/7_ | Visual analysis, Activity amount (dosimetry) | - | - |
| Liu et al. (2023) (139) | Breast cancer  (n = 5) | ^18^F-AlF-NOTA-HER2-BCH or ^18^F-AlF-RESCA-HER2-BCH (Affibody, HER2) | 231.29 MBq, 1mg | 2, 4 h.  1 patient additionally 0-45 min | SUV_mean,_ SUV_max,_ Tissue-to-background ratio | - | - |
| Zhou et al. (2021) (140) | Gastric cancer (n = 32) | ^68^Ga-NOTA-MAL-MZHER2 (Affibody, HER2) | 3.7 MBq/kg | 1, 2, 3 h (n=13), 2, 4 h (n=2), 2 h (n=17) | Activity concentration  (healthy tissues)  SUV_max_ (lesions) | SUV_max_ correlated with  biopsy | Baseline SUV_max_ higher in lesions with response than in those without response |
| Miao et al. (2022) (141) | Breast cancer  (n = 24) | ^68^Ga-NOTA-MAL-MZHER2 (Affibody, HER2) | 3.0 MBq/kg | 2 h | SUV_max_ (lesions) | Sensitivity 91.7%. Specificity 84.6%) | - |
| Baum et al. (2010) (142) | Breast cancer  (n = 3) | ^68^Ga-ABY-002 (Affibody, HER2) | 110-267 MBq, ~80-90 μg | 90/95/135/250 min | SUV | - | - |
| Beylergil et al. (2013) (143) | Breast cancer  (n = 15) | ^68^Ga-DOTA-  F(ab’)2 –trastuzumab (HER2) | 236 MBq, 4.5–5.5 mg | 1.1, 1.8, 2.7 h | SUV_mean_ (healthy tissues),  SUV_max_ (small healthy tissues) | - | - |
| Sandström et al. (2016) (144) | Breast cancer  (n = 8) | ^68^Ga-ABY-025 (Affibody, HER2) | 215/241 MBq, 78/427 μg | 1, 2,4 h | SUV_mean_ (healthy tissues) | - | - |
| Sörensen et al. (2016) (145) | Breast cancer  (n = 16) | ^68^Ga-ABY-025 (affibody, HER2) | 212 MBq, 78/427 μg | 1, 2, 4 h | SUV_max_ (lesions) | SUV correlated with biopsy HER2-scores | - |
| Wang et al. (2022) (146) | Lung cancer  (n = 3) | ^68^Ga-NODAGA-SNA006 (nanobody, CD8+) | 164 MBq, 100/800 μg | 15-30, 60-90, 120 min | SUV_max,_  SUV_mean_ | Significant correlation between tumor CD8 expression and PET uptake | - |
| Gondry et al. (2023) (147) | Solid tumors  (n = 7) | ^68^Ga-NOTA-anti-CD206-sdAb (sdAb, CD206) | 191 MBq, 58.2-96.8 μg | 11, 90, 150 min | SUV_mean_ (healthy tissues)  SUV_max_ (lesions) | - | - |
| Keyaerts et al. (2016) (148) | HER2+ breast cancer (n = 20) | ^68^Ga-HER2-nanobody | 107 MBq, 0.-01/0.1/1.0 mg | 10, 60, 90 min | SUV_mean_ | - | - |
| Li et al. (2023) (149) | Colorectal cancer (n = 9) | ^68^Ga-HNI01 (nanobody, CEA) | 151 MBq, 0.2 mg | Dynamic 0-40 min, 2 h | SUV_max_ | - | - |
| Natarajan et al. (2020) (157) | Ovarian (n = 1) and breast cancer (n = 1) | ^64^Cu-Bfab (Fab, CA6) | 204/432 MBq, 0.25/0.38 mg | 1 h, D_1_ | SUV | - | - |

**Supplementary Table 3.** Overview of clinical immunoPET publications extending beyond single time point semi-quantitative analysis and including tracer-kinetic and pharmacokinetic modelling approaches.

| Publication (year) (reference number in main manuscript) | Study cohort | Tracer (type, target) | Average Radioactivity; Total protein doses (co-injection) or preload | Imaging protocol | Quantification methods | Correlation to Biopsies / tumor resections | Correlation to Response |
| --- | --- | --- | --- | --- | --- | --- | --- |
| Jauw et al. (2019) (158) | CD44-expressing solid tumors (n = 13) | ^89^Zr-RG7356 (CD44) | 37 MBq, 1/100/200,450/675 mg | 1 h, D_1_, D_4_ | TBR AUC ratios (healthy tissues)  SUV_peak_, TBR AUC ratios (lesions) | - | - |
| Jauw et al. (2019) (159) | Data of 4 ^89^Zr-mAb tracers: obinutuzumab (CD20) (160), cetuximab (EGFR) (108), huJ951 (PSMA) (83) and trastuzumab (HER2) (85) | | | | Patlak modeling (healthy tissues) | - | - |
| Miedema et al. (2023) (162) | Head and neck (n = 2) and lung cancer (n = 4), on anti-PD-1 treatment | ^89^Zr-BI 754111 (mAb, LAG3) | 37 MBq, 4 mg. Second scan with additional 40 or 600 mg cold dose. | 2 h, D_4_, D_6_ | SUV_mean_, Patlak modeling (healthy tissues);  Tumor-to-plasma ratio, SUV_peak_ (lesions) | Tumor-to-plasma ratios correlated with immune cell-derived RNA signatures | - |
| Miedema et al. (2023) (163) | Data of five ^89^Zr-mAb studies (nivolumab, pembrolizumab, durvalumab, BI 754111 and ipilimumab) | | 37 MBq, 2-3 different mass doses for each mAb | At least two time points 24 h p.i. | Patlak modeling (healthy tissues) | - | - |
| Menke-van der Houven van Oordt et al. (2019) (164) | HER3+ cancer (n = 6) | ^89^Zr-GSK284933 (mAb, HER3) | 38 MBq, 8/24 mg tracer at baseline, 24-30 mg/kg before second tracer at repeat scan | First 3 patients at baseline: 2 h, D_2_, D_5_ at baseline. All other scans: D_2_, D_5_ | SUV_mean_, Patlak modeling (healthy tissues), SUV_peak_ (lesions) | - | No correlation between uptake and response |
| Wijngaarden et al. (2023) (39) | Data of two studies above: ^89^Zr-cetuximab(EGFR) (108) and ^89^Zr-GSK2849330(EGFR) (164) | | | | Patlak modeling, SUV_peak_, TBR_peak_, tumor-to-plasma_peak_ | - | - |
| Daghighian et al. (1993) (165) | Glioma (n = 1) | ^124^I-3F8 (mAb, GD2 ganglioside) | 96.2 MBq | 0-90 min, 18-19 h, D_3_, D_10_, D11 | 2-tissue compartment modeling | - | - |
| Zanzonico et al. (2015) (116) | Colorectal cancer (n = 11),  Data from (47) | ^124^I-huA33 (mAb, A33) | Approx. 370 MBq, 10 mg | 3 h, D_2_, D_5_-D_9_ | Nonlinear compartment modeling | Agreement of antigen concentration from model fitting with in vitro binding studies with resected tumor specimens | - |
| Omidvari et al. (2023) (17) | COVID-19 (n = 5)  Control (n = 3) | ^89^Zr-IAB22M2C (minibody, CD8+) | 18.8 MBq, 1.5 mg | 90 min dynamic scan at injection, 60 min static scans at 6 h and D2 | 1-tissue and 2-tissue compartment modeling, Patlak modeling, SUV_mean_, SUV_peak_ (lymph nodes), tissue-to-blood ratios | - | - |
| Alhuseinalkhudhur et al. (2020) (166) | Breast cancer (n = 16) | ^68^Ga-ABY-025 (affibody, HER2) | 241 MBq, 0.427 mg | 45 min dynamic scan at injection | 1-tissue and 2-tissue compartment modeling, SUV_mean_ | . | - |
